# Global, regional, and national burden of migraine and tension-type headache, 1990–2016: a systematic analysis for the Global Burden of Disease Study 2016

**DOI:** 10.1016/S1474-4422(18)30322-3

**Published:** 2018-11

**Authors:** GBD 2016 Headache Collaborators, Lars Jacob Stovner, Emma Nichols, Timothy J Steiner, Foad Abd-Allah, Ahmed Abdelalim, Rajaa M Al-Raddadi, Mustafa Geleto Ansha, Aleksandra Barac, Isabela M Bensenor, Linh Phuong Doan, Dumessa Edessa, Matthias Endres, Kyle J Foreman, Fortune Gbetoho Gankpe, Gururaj Gopalkrishna, Alessandra C Goulart, Rahul Gupta, Graeme J Hankey, Simon I Hay, Mohamed I Hegazy, Esayas Haregot Hilawe, Amir Kasaeian, Dessalegn H Kassa, Ibrahim Khalil, Young-Ho Khang, Jagdish Khubchandan, Yun Jin Kim, Yoshihiro Kokubo, Mohammed A Mohammed, Maziar Moradi-Lakeh, Huong Lan Thi Nguyen, Yirga Legesse Nirayo, Mostafa Qorbani, Anna Ranta, Kedir T Roba, Saeid Safiri, Itamar S Santos, Maheswar Satpathy, Monika Sawhney, Mekonnen Sisay Shiferaw, Ivy Shiue, Mari Smith, Cassandra E I Szoeke, Nu Thi Truong, Narayanaswamy Venketasubramanian, Kidu gidey weldegwergs, Ronny Westerman, Tissa Wijeratne, Bach Xuan Tran, Naohiro Yonemoto, Valery L Feigin, Theo Vos, Christopher J L Murray

## Abstract

**Background:**

Through the Global Burden of Diseases, Injuries, and Risk Factors (GBD) studies, headache has emerged as a major global public health concern. We aimed to use data from the GBD 2016 study to provide new estimates for prevalence and years of life lived with disability (YLDs) for migraine and tension-type headache and to present the methods and results in an accessible way for clinicians and researchers of headache disorders.

**Methods:**

Data were derived from population-based cross-sectional surveys on migraine and tension-type headache. Prevalence for each sex and 5-year age group interval (ie, age 5 years to ≥95 years) at different time points from 1990 and 2016 in all countries and GBD regions were estimated using a Bayesian meta-regression model. Disease burden measured in YLDs was calculated from prevalence and average time spent with headache multiplied by disability weights (a measure of the relative severity of the disabling consequence of a disease). The burden stemming from medication overuse headache, which was included in earlier iterations of GBD as a separate cause, was subsumed as a sequela of either migraine or tension-type headache. Because no deaths were assigned to headaches as the underlying cause, YLDs equate to disability-adjusted life-years (DALYs). We also analysed results on the basis of the Socio-demographic Index (SDI), a compound measure of income per capita, education, and fertility.

**Findings:**

Almost three billion individuals were estimated to have a migraine or tension-type headache in 2016: 1·89 billion (95% uncertainty interval [UI] 1·71–2·10) with tension-type headache and 1·04 billion (95% UI 1·00–1·09) with migraine. However, because migraine had a much higher disability weight than tension-type headache, migraine caused 45·1 million (95% UI 29·0–62·8) and tension-type headache only 7·2 million (95% UI 4·6–10·5) YLDs globally in 2016. The headaches were most burdensome in women between ages 15 and 49 years, with migraine causing 20·3 million (95% UI 12·9–28·5) and tension-type headache 2·9 million (95% UI 1·8–4·2) YLDs in 2016, which was 11·2% of all YLDs in this age group and sex. Age-standardised DALYs for each headache type showed a small increase as SDI increased.

**Interpretation:**

Although current estimates are based on limited data, our study shows that headache disorders, and migraine in particular, are important causes of disability worldwide, and deserve greater attention in health policy debates and research resource allocation. Future iterations of this study, based on sources from additional countries and with less methodological heterogeneity, should help to provide stronger evidence of the need for action.

**Funding:**

Bill & Melinda Gates Foundation.

## Introduction

Migraine and other headache disorders are among the most prevalent disorders worldwide,^1^ but recognition of their importance for public health has come only since 2000. This delay has occurred in part because headache is not fatal and does not result in permanent or objective disability, and in part because headaches are experienced by most people from time to time, which has hindered the realisation that headache disorders are debilitating for a relatively large minority of the people who are affected.

The Global Burden of Diseases, Injuries, and Risk Factors (GBD) studies have as one of their main aims the evaluation not only of mortality but also of non-fatal health outcomes. GBD offers a method of quantifying health loss in time units, enabling comparisons over time and across conditions, cultures, and countries, and, from 2016, at subnational levels in some countries. GBD has become an important tool for priority setting and planning of health services by international health organisations and governments.

Migraine was not included in GBD 1990, but was added in GBD 2000. In GBD 2010, tension-type headache was added and in GBD 2013, medication overuse headache was included. In GBD 2000, migraine was ranked as the 19th cause of disability globally.^2^ For the GBD 2000 study, data were absent for more than half of the world's population. When new data came from big countries like Russia, China, India, and some parts of Africa, and with tension-type headache and medication overuse headache also taken into account, headache disorders were collectively the third cause of disability in people under 50 years of age in GBD 2015.^3^ Since GBD 2010, prevalence and burden of disability have been re-estimated for the full time period from 1990 until the most recent year for which data are available, each time incorporating new data sources and any updates to methods.

**Research in context**

**Evidence before this study**

Since 2000, the Global Burden of Diseases, Injuries, and Risk Factors (GBD) studies have produced estimates of prevalence and burden of migraine. Since GBD 2010, tension-type headache and medication overuse headache have been added and estimates have been made by country spanning the period from 1990 to the most recent year for which data are available. Headache disorders, and in particular, migraine, have been found to be highly prevalent and a cause of large burden. To date, no research article has focused on the detailed methods and results of headache estimates from GBD. With the present study, we updated a previous systematic review covering 1980–2001 by doing a review that searched PubMed for articles using the terms “migraine”, “tension”, “headache”, “medication”, and “epidemiology” from Jan 1, 2001, until Dec 31, 2015. There were no language restrictions.

**Added value of this study**

In 2016, of all GBD causes of disease, tension-type headache was the third most prevalent, and migraine the sixth. In terms of years of life lived with disability, migraine ranked second globally, and was among the ten most disabling disorders in each of the 21 GBD regions. It was particularly burdensome among young and middle-aged women. Unlike many other diseases and injuries quantified in GBD studies, headache showed no clear relation to sociodemographic development, as measured by the Socio-demographic Index. No risk factors have yet been established in the GBD studies for headache disorders, and headache epidemiological studies are absent in many countries and regions.

**Implications of all the available evidence**

Through the GBD studies, headache disorders, and in particular, migraine, have been shown to be among the most disabling disorders worldwide. Many fatal and disabling disorders decrease with socioeconomic development, but this does not seem to be true for migraine and tension-type headache. Hence, their relative importance is likely to increase in the future. More high-quality headache epidemiological studies and studies aiming to identify modifiable risk factors should be done. Effective strategies to modify the course of headaches and alleviate pain exist, but many people affected by headache are not benefiting from this knowledge.

Given the importance of headache disorders for global public health, which has become evident through GBD, we wanted to inform an audience of headache specialists about these studies. The aims of the present Article are to provide an overview of the GBD methods as applied to headache, to present detailed results of the update for 1990–2016 on headache burden in different world regions and with time trends, and to discuss the implications of these results both for future iterations of GBD and for health policies around the world.

## Methods

### Overview

The main elements of the GBD methods, both general and pertaining to migraine and tension-type headache, are described in the appendix. In the main text of this Article, we concentrate on methods pertaining to estimation of the burden of migraine and tension-type headache. A flowchart of the different steps in these methods is shown in the appendix.

In the GBD cause hierarchy, migraine and tension-type headache are individual disorders on Level 3, under neurological disorders (Level 2) and non-communicable diseases (Level 1). No further subdivision exists for headaches, so each reappears at Level 4. In GBD 2013 and GBD 2015, medication overuse headache was treated as a separate disorder, but in GBD 2016 it was considered a sequela of either migraine or tension-type headache. The burden of medication overuse headache was therefore added to the burdens estimated for these headache types according to a meta-analysis of three studies reporting the proportions of medication overuse headache resulting from migraine (73·4%, 95% uncertainty interval [UI] 63·9–82·0) or tension-type headache (26·6%, 18·0–36·1).^4^, ^5^, ^6^

In GBD, disease burden is estimated in disability-adjusted life-years (DALYs), which are the sum of years of life lost (YLLs) to premature mortality and years of life lived with disability (YLDs). Because GBD does not estimate any deaths from headache disorders as the underlying cause, DALYs for headaches are equivalent to YLDs. YLDs for each headache disorder are calculated from its prevalence and the mean time patients spend with that type of headache multiplied by the associated disability weight. The determination of headache disability weights through population and internet surveys was on the basis of lay descriptions (appendix).^7^, ^8^ The disability weight for migraine was 0·434, meaning that during an attack the affected person experiences health loss of 43·4% compared with a person in full health. The disability weight for medication overuse headache was 0·223 and for tension-type headache was 0·037. After all diseases were estimated separately, an adjustment was made to YLDs to account for comorbidity by use of simulation methods assuming a multiplicative, rather than additive, model. This adjustment led to a downward correction for YLDs for migraine in women and children by factors ranging from 2·1% (at ages 5–9 years) to 20·6% (at ages ≥95 years), reflecting a strong correlation between comorbidity and age. The corresponding figures in males were 2·1% and 20·7%, respectively.

### Data sources

For headaches, the data sources were mostly published population-based studies of prevalence; however, survey data for which we had the individual record data were also included. The PubMed search terms are shown in the appendix. Reference lists in published articles were reviewed and data were solicited from our GBD network of more than 2500 collaborators.

Only studies diagnosing the headaches according to the International Classification of Headache Disorders (ICHD) were considered. This classification was first published in 1988, updated in 2004 (ICHD-2),^9^ and updated again in 2018 (ICHD-3).^10^ In the three ICHD versions, no major differences exist with regard to diagnostic criteria of migraine, tension-type headache, and medication overuse headache. For evaluation of headache burden, these diagnoses were selected because they are, by far, the most common and of the greatest public health importance.^11^ Cluster headache causes severe pain and disability, but with a lifetime prevalence of approximately 0·2% the effect on public health is much less.^1^ Diagnosis of the many secondary headaches (eg, due to infections or brain tumours) is difficult in epidemiological studies, and the burden of these headaches should be attributed to the underlying disorder.^11^ An exception is secondary headache caused by medication overuse, which occurs almost exclusively in patients with either migraine or tension-type headache.

For GBD 2016, data on migraine were extracted from 135 studies, covering 16 of the 21 GBD world regions. For tension-type headache, data were extracted from 76 studies covering 16 GBD regions. All data sources are available online. For medication overuse headache, data were extracted from 37 studies from ten regions (for details about the numbers of studies from each region see the appendix). In addition, hospital claims data from the USA on migraine and tension-type headache for 3 years (2000, 2010, and 2012) were used.^12^, ^13^, ^14^ Sets of claims data were included because, owing to their size, they provide more detailed information on age patterns and time trends than published epidemiological studies, and also provide estimates on subnational locations. Because claims data might not be nationally representative, do not refer to the ICHD criteria, and capture only those individuals who are able to seek health care, they are evaluated for systematic biases compared with data sources of high quality (appendix).

Approximately half of the 135 studies from which data were extracted were from three of the 21 GBD regions (western Europe, high-income North America, and high-income Asia Pacific), and no data on any of the headaches were available from the Caribbean, central sub-Saharan Africa, southern sub-Saharan Africa, or Oceania (see appendix) regions. In all regions and countries, prevalence was estimated with a Bayesian meta-regression model (DisMod-MR 2.1), and estimates were obtained in this way also for countries and regions where no relevant headache studies had been done.

### Calculation of proportion of time in the symptomatic state

Headache disorders are modelled as chronic episodic conditions. The prevalence reflects the individuals in the population who have had at least one episode in the past 12 months fulfilling ICHD criteria. To calculate the average proportion of time with headache (ie, in the symptomatic state) necessary for YLD calculation, 13 population-based studies were identified that had data on frequency and duration of migraine attacks (appendix). From these studies, we estimated the average number of hours migraineurs spend in attacks, and expressed this as a proportion of a year, which was 8·5% (95% UI 5·8–11·2)**.**

For tension-type headache, seven studies on duration and frequency of attacks showed that affected people spend, on average, 4·7% (95% UI 1·3–8·0) of their time with headache. For both migraine and tension-type headache, frequency and duration were reported most commonly in categories, and the midpoint was assumed to represent each category. For medication overuse headache, only one study included in GBD 2016 (from Russia) gave data on time in symptomatic state, reporting a mean headache frequency of 23·1 (SD 6·7) days per month.^15^ According to the ICHD-3 definition, medication overuse headache is present on more than 15 days per month for more than 3 months.^10^

### Modelling of prevalence

In the mathematical modelling, the mortality due to headache was set at zero,^16^ as was occurrence below age 5 years.^17^ In the sources used for this study, prevalence rates vary, but the degree to which this reflects real variation across borders and time, or methodological differences, is mostly not known. Method most probably plays a large role because results can be substantially influenced by relatively minor differences, such as variations in the screening question.^18^ To adjust for differences in methodological quality, all prevalence studies included in GBD are scored according to a modified version (dichotomised variables) of published methodological quality criteria for headache epidemiological studies,^11^ taking into account the representativeness of the population of interest (representative of country or community *vs* selected population), quality of sampling (random sample of the population of interest *vs* not random sample), recall period (1-year prevalence *vs* other recall period), participation rate (≥70% *vs* <70%), survey method (face to face with headache expert or trained interviewer *vs* other), validation of diagnostic instrument (sensitivity or specificity ≥70% *vs* <70% or no validation), and application of ICHD criteria (strict criteria or reasonable modification of criteria *vs* other modification of criteria). In DisMod-MR 2.1, these methodological variables were evaluated for a systematic difference and corrected accordingly (appendix).

### Socio-demographic Index (SDI)

We examined the relationships between migraine and tension-type headache DALYs and SDI, a composite measure of income per capita, education, and fertility.^19^ We also present results by groupings of countries into quintiles (high SDI, middle-high SDI, middle SDI, middle-low SDI, and low SDI) based on their 2016 SDI value. Additional details on the SDI methods can be found online.

### Role of the funding source

The funder of the study had no role in study design, data collection, data analysis, data interpretation or the writing of the report. All authors had full access to the data in the study and had final responsibility for the decision to submit for publication.

## Results

**Figure 1:**
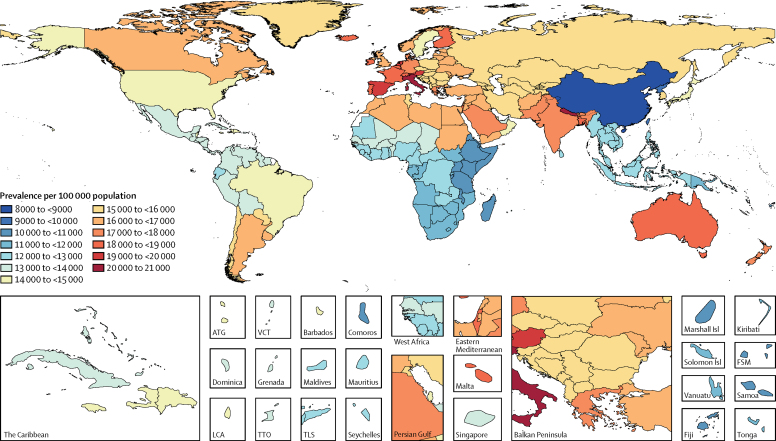
Age-standardised prevalence of migraine per 100 000 population by location for both sexes, 2016 ATG=Antigua and Barbuda. FSM=Federated States of Micronesia. LCA=Saint Lucia. TLS=Timor-Leste. TTO=Trinidad and Tobago. VCT=Saint Vincent and the Grenadines.

**Figure 2:**
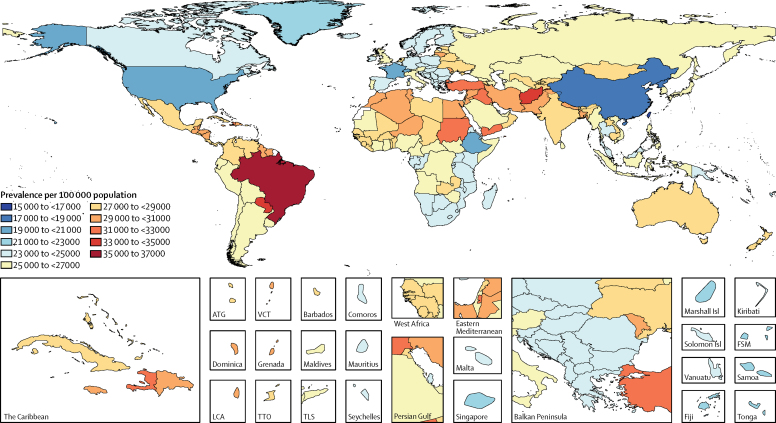
Age-standardised prevalence of tension-type headache per 100 000 population by location for both sexes, 2016 ATG=Antigua and Barbuda. FSM=Federated States of Micronesia. LCA=Saint Lucia. TLS=Timor-Leste. TTO=Trinidad and Tobago. VCT=Saint Vincent and the Grenadines.

In 2016, almost three billion individuals were estimated to have a headache disorder: 1·89 billion (95% UI 1·71–2·10) with tension-type headache and 1·04 billion (95% UI 1·00–1·09) with migraine (table). All data on age-standardised prevalence and YLDs for all countries and regions, for both 1990 and 2016, are publicly available in online visualisations and results download tools. For tension-type headache, the global age-standardised prevalence was 26·1% (23·6–29·0) overall: 30·8% (28·0–34·0) for women and 21·4% (19·2–23·9) for men. All results for both sexes are publicly available online. For migraine, global age-standardised prevalence was 14·4% (13·8–15·0) overall: 18·9% (18·1–19·7) for women, and 9·8% (9·4–10·2) for men. Maps of age-standardised prevalence of migraine and tension-type headache in each country are shown in Figure 1, Figure 2. In 2016, the age-standardised prevalence of migraine was highest in Italy and Nepal, and that of tension-type headache was highest in Brazil and Afghanistan. The lowest prevalence of both disorders was in China.

**Table:** Prevalent cases and YLDs for migraine and tension-type headache in 2016 and percentage change of age-standardised rates by location

	**Migraine**				**Tension-type headache**			
	Prevalence		YLDs		Prevalence		YLDs	
	2016 counts	Percentage change in age-standardised rates, 1990–2016	2016 counts	Percentage change in age-standardised rates, 1990–2016	2016 counts	Percentage change in age-standardised rates, 1990–2016	2016 counts	Percentage change in age-standardised rates, 1990–2016
**Global**	**1 044 771 478 (999 534 692 to 1 087 968 951)**	**−1·8% (−2·0 to −1·5)**	**45 121 909 (29 045 835 to 62 826 904)**	**−0·2% (−0·8 to 0·4)**	**1 890 670 389 (1 707 786 493 to 2 097 761 629)**	**−7·3% (−7·8 to −6·7)**	**7 195 122 (4 614 628 to 10 499 903)**	**−0·2% (−2·5 to 1·9)**
High SDI	167 752 331 (162 068 750 to 173 328 886)	−2·4% (−3·0 to −1·8)	7 183 304 (4 631 325 to 10 020 672)	−1·6% (−2·3 to −0·9)	245 115 740 (226 317 507 to 265 077 769)	−7·1% (−8·7 to −5·3)	1 055 366 (679 220 to 1 539 885)	−1·9% (−3·8 to −0·1)
High-middle SDI	172 643 687 (165 086 497 to 180 178 966)	−5·1% (−5·7 to −4·5)	7 760 262 (5 041 528 to 10 735 182)	−3·4% (−4·4 to −2·2)	307 673 576 (277 323 460 to 341 968 784)	−8·8% (−9·7 to −7·9)	1 327 611 (851 252 to 1 948 910)	−2·5% (−4·8 to −0·3)
Middle SDI	294 085 908 (281 017 554 to 306 959 499)	2·9% (2·5 to 3·2)	12 911 188 (8 334 437 to 17 962 205)	4·6% (3·8 to 5·4)	569 499 609 (511 283 994 to 635 895 815)	−6·1% (−6·8 to −5·5)	2 160 117 (1 381 284 to 3 171 241)	2·9% (−0·1 to 5·9)
Low-middle SDI	329 933 660 (315 287 837 to 344 134 051)	−2·1% (−2·6 to −1·7)	13 869 352 (8 882 881 to 19 370 615)	0·0% (−0·9 to 0·9)	596 330 852 (536 364 468 to 666 261 088)	−11·0% (−12·0 to −10·0)	2 096 630 (1 328 963 to 3 104 125)	−0·9% (−4·1 to 2·6)
Low SDI	84 126 809 (79 807 328 to 88 407 248)	−2·2% (−2·8 to −1·6)	3 546 725 (2 273 280 to 5 001 221)	0·0% (−1·1 to 1·0)	175 779 968 (157 143 060 to 197 823 486)	−8·2% (−9·1 to −7·3)	572 499 (361 398 to 852 525)	−0·6% (−3·2 to 2·3)
**High-income North America**	**53 344 669 (52 296 498 to 54 399 071)**	**−2·8% (−3·9 to −1·6)**	**2 276 046 (1 465 260 to 3 185 259)**	**−2·1% (−3·3 to −0·9)**	**74 430 873 (71 702 414 to 76 995 483)**	**−10·6% (−15·5 to −5·3)**	**329 298 (211 947 to 477 734)**	**−3·6% (−7·2 to −0·8)**
Canada	6 301 113 (6 085 757 to 6 517 308)	−8·3% (−10·2 to −6·3)	264 844 (168 731 to 371 278)	−6·8% (−9·8 to −3·5)	8 696 062 (7 792 089 to 9 694 541)	−19·1% (−22·6 to −15·8)	36 169 (23 180 to 53 026)	−7·8% (−13·1 to −1·9)
Greenland	8107 (7698 to 8523)	−3·7% (−5·9 to −1·4)	338 (218 to 475)	−2·5% (−5·7 to 1·1)	11 661 (10 384 to 13 064)	−14·4% (−17·3 to −11·2)	46 (29 to 69)	−5·0% (−10·1 to 0·8)
USA	47 016 985 (46 100 523 to 47 899 333)	−2·0% (−3·3 to −0·6)	2 010 075 (1 296 354 to 2 814 841)	−1·4% (−2·8 to −0·1)	65 697 424 (63 755 143 to 67 595 782)	−9·3% (−15·1 to −3·1)	292 968 (188 787 to 423 703)	−3·1% (−6·8 to −0·2)
**Australasia**	**5 334 994 (5 091 834 to 5 583 436)**	**−1·1% (−3·0 to 1·1)**	**219 745 (139 957 to 309 573)**	**−0·5% (−3·1 to 2·4)**	**8 124 243 (7 342 759 to 9 000 939)**	**−4·1% (−6·3 to −1·6)**	**29 676 (18 804 to 43 770)**	**−0·7% (−4·6 to 3·9)**
Australia	4 543 179 (4 330 318 to 4 756 061)	−1·3% (−3·5 to 1·3)	186 837 (119 136 to 263 360)	−0·6% (−3·7 to 2·8)	6 841 911 (6 181 717 to 7 597 540)	−4·1% (−6·6 to −1·1)	25 009 (15 828 to 36 960)	−0·7% (−5·2 to 4·7)
New Zealand	791 815 (754 681 to 830 074)	−0·4% (−2·9 to 2·2)	32 908 (20 979 to 46 303)	0·0% (−3·2 to 3·5)	1 282 332 (1 162 388 to 1 415 407)	−3·9% (−6·9 to −0·8)	4667 (2954 to 6859)	−0·6% (−5·1 to 4·5)
**High-income Asia Pacific**	**27 032 727 (25 957 086 to 28 086 345)**	**−1·8% (−2·6 to −1·0)**	**1 137 019 (727 818 to 1 585 857)**	**−1·4% (−2·6 to −0·2)**	**49 382 811 (44 532 535 to 54 333 934)**	**−1·0% (−2·3 to 0·4)**	**173 818 (110 645 to 255 402)**	**0·0% (−1·8 to 1·8)**
Brunei	67 038 (63 064 to 70 690)	−0·9% (−3·3 to 1·7)	2782 (1768 to 3935)	0·0% (−3·4 to 3·4)	115 076 (102 272 to 128 531)	−1·9% (−4·9 to 1·2)	391 (244 to 583)	0·7% (−4·1 to 5·9)
Japan	17 763 393 (17 076 332 to 18 452 854)	−1·4% (−2·1 to −0·9)	753 131 (484 557 to 1 049 616)	−1·3% (−2·2 to −0·3)	33 825 326 (30 555 799 to 37 158 324)	−0·4% (−1·2 to 0·3)	119 701 (76 091 to 175 096)	0·0% (−1·2 to 1·3)
Singapore	577 459 (549 744 to 604 482)	−2·0% (−4·1 to 0·6)	24 637 (15 855 to 34 643)	−0·9% (−4·4 to 2·6)	966 159 (866 174 to 1 077 672)	−1·4% (−4·9 to 2·4)	3641 (2311 to 5365)	0·7% (−4·2 to 6·3)
South Korea	8 624 838 (8 242 808 to 9 009 884)	−4·1% (−6·3 to −2·1)	356 469 (226 037 to 498 464)	−2·9% (−5·9 to 0·3)	14 476 250 (13 005 907 to 16 000 912)	−2·1% (−5·9 to 2·7)	50 085 (31 775 to 74 160)	0·1% (−4·7 to 5·4)
**Western Europe**	**80 808 422 (77 634 593 to 84 034 957)**	**−1·2% (−2·0 to −0·5)**	**3 447 004 (2 225 424 to 4 805 927)**	**−0·2% (−1·3 to 0·9)**	**107 733 424 (97 057 256 to 119 221 162)**	**−5·7% (−6·8 to −4·6)**	**484 810 (308 046 to 714 229)**	**0·1% (−2·2 to 2·5)**
Andorra	15 188 (14 355 to 16 017)	−1·3% (−3·6 to 1·3)	658 (427 to 914)	−0·6% (−3·7 to 2·7)	19 967 (17 952 to 22 290)	−4·2% (−7·6 to −0·9)	96 (61 to 145)	−0·5% (−5·4 to 4·9)
Austria	1 819 880 (1 746 912 to 1 893 539)	−1·0% (−3·0 to 1·0)	77 370 (50 017 to 108 066)	−0·3% (−3·2 to 2·5)	2 282 375 (2 039 267 to 2 542 347)	−3·9% (−6·9 to −0·6)	10 546 (6626 to 15 484)	−0·2% (−5·1 to 5·3)
Belgium	2 124 832 (2 013 452 to 2 239 948)	−1·2% (−3·8 to 1·3)	91 868 (59 314 to 127 901)	−0·7% (−3·9 to 2·8)	2 822 458 (2 532 967 to 3 130 995)	−4·5% (−7·5 to −1·4)	13 345 (8423 to 19 937)	−0·4% (−5·4 to 5·8)
Cyprus	183 856 (173 763 to 194 234)	−1·1% (−3·6 to 1·4)	7892 (5 088 to 11 079)	0·2% (−3·1 to 3·8)	243 221 (217 611 to 270 212)	−6·8% (−9·7 to −3·5)	1108 (696 to 1647)	0·9% (−4·3 to 7·1)
Denmark	1 026 797 (974 682 to 1 074 089)	−1·0% (−3·5 to 1·4)	44 524 (28 840 to 62 190)	−0·1% (−3·2 to 3·2)	1 409 715 (1 266 690 to 1 575 454)	−3·9% (−6·8 to −0·9)	6639 (4268 to 9636)	1·5% (−3·2 to 6·5)
Finland	1 012 067 (955 827 to 1 065 559)	−1·8% (−4·2 to 0·8)	43 829 (28 398 to 61 020)	−0·8% (−4·1 to 2·6)	1 351 804 (1 212 022 to 1 501 509)	−5·1% (−7·9 to −1·9)	6450 (4109 to 9646)	−0·5% (−5·3 to 4·9)
France	11 892 970 (11 406 938 to 12 378 827)	−0·4% (−2·8 to 2·1)	517 500 (332 963 to 715 766)	0·7% (−2·4 to 4·2)	13 357 050 (11 965 824 to 14 888 605)	−3·0% (−6·4 to 0·7)	71 795 (45 872 to 103 336)	2·0% (−3·0 to 7·8)
Germany	14 730 968 (14 105 025 to 15 352 755)	−1·5% (−3·8 to 0·7)	620 732 (401 735 to 869 239)	−0·8% (−4·0 to 2·5)	20 673 287 (18 543 280 to 22 985 159)	−7·8% (−10·8 to −4·6)	87 477 (55 662 to 128 289)	−1·9% (−6·9 to 3·5)
Greece	2 014 931 (1 917 185 to 2 108 577)	−1·6% (−3·8 to 0·7)	87 918 (57 132 to 122 557)	−0·7% (−3·6 to 2·4)	2 669 218 (2 395 448 to 2 964 387)	−5·0% (−7·9 to −2·0)	12 997 (8196 to 19 424)	0·1% (−5·5 to 5·8)
Iceland	61 873 (58 419 to 65 240)	−1·1% (−3·6 to 1·6)	2671 (1710 to 3752)	−0·3% (−3·4 to 3·2)	81 725 (73 517 to 90 858)	−5·0% (−8·1 to −1·7)	382 (241 to 574)	0·1% (−4·9 to 5·9)
Ireland	880 498 (832 357 to 928 391)	−1·2% (−3·6 to 1·5)	37 863 (24 481 to 53 165)	−0·1% (−3·3 to 3·3)	1 154 239 (1 036 694 to 1 286 283)	−6·5% (−9·6 to −3·2)	5375 (3396 to 8140)	0·4% (−5·1 to 7·0)
Israel	1 457 632 (1 379 341 to 1 535 790)	−1·4% (−3·6 to 1·2)	62 150 (40 143 to 87 951)	−0·6% (−3·8 to 3·0)	2 010 964 (1 798 842 to 2 248 648)	−4·8% (−7·7 to −1·6)	8642 (5377 to 12 912)	−0·3% (−5·2 to 5·1)
Italy	12 977 731 (12 486 461 to 13 468 642)	−0·6% (−2·7 to 1·6)	534 275 (343 210 to 753 112)	0·1% (−2·8 to 3·3)	16 789 700 (15 107 193 to 18 628 319)	−5·8% (−8·8 to −2·7)	67 781 (43 106 to 99 628)	−0·8% (−6·0 to 5·0)
Luxembourg	122 651 (117 652 to 127 560)	−0·8% (−3·0 to 1·5)	5205 (3338 to 7292)	0·3% (−2·8 to 3·7)	157 274 (141 189 to 174 944)	−4·6% (−7·8 to −1·4)	713 (462 to 1039)	0·8% (−4·0 to 6·3)
Malta	80 226 (75 934 to 84 851)	−3·4% (−5·8 to −1·0)	3477 (2270 to 4898)	−2·0% (−5·4 to 1·1)	108 621 (97 601 to 120 997)	−6·9% (−9·9 to −3·8)	508 (319 to 764)	−0·6% (−5·5 to 5·1)
Netherlands	3 489 189 (3 341 633 to 3 621 037)	−0·4% (−2·4 to 1·6)	142 336 (90 809 to 199 718)	0·2% (−2·8 to 3·2)	4 834 235 (4 344 123 to 5 369 211)	−4·2% (−7·1 to −0·8)	18 232 (11 610 to 26 839)	−0·1% (−4·7 to 5·1)
Norway	895 018 (855 272 to 933 252)	−1·2% (−3·4 to 1·1)	37 923 (24 456 to 53 206)	0·4% (−2·9 to 3·8)	1 340 876 (1 205 023 to 1 497 829)	−8·7% (−15·2 to −3·1)	5410 (3445 to 7870)	0·1% (−6·1 to 7·2)
Portugal	2 051 173 (1 936 439 to 2 165 133)	−1·5% (−3·9 to 1·0)	88 374 (57 161 to 124 408)	−0·3% (−3·7 to 3·3)	2 783 841 (2 499 031 to 3 090 289)	−6·2% (−9·2 to −2·8)	12 847 (8150 to 19 223)	0·2% (−4·8 to 5·8)
Spain	9 449 914 (9 072 573 to 9 842 296)	−1·7% (−3·7 to 0·2)	405 466 (261 007 to 570 711)	−0·5% (−3·5 to 2·8)	11 981 430 (10 665 454 to 13 301 336)	−6·5% (−9·4 to −3·2)	56 193 (35 172 to 83 084)	0·6% (−4·7 to 6·5)
Sweden	1 487 390 (1 431 549 to 1 543 234)	−2·0% (−3·7 to −0·3)	65 352 (42 366 to 90 761)	−1·1% (−3·5 to 1·6)	2 422 653 (2 155 615 to 2 715 005)	−4·3% (−6·9 to −2·0)	10 567 (6700 to 15 201)	0·2% (−3·4 to 4·2)
Switzerland	1 610 830 (1 540 457 to 1 682 449)	−1·3% (−3·4 to 0·9)	69 839 (45 338 to 98 043)	−0·4% (−3·5 to 2·9)	1 832 194 (1 633 381 to 2 053 349)	−1·5% (−5·3 to 3·4)	9709 (6146 to 14 649)	0·5% (−4·4 to 6·0)
UK	11 340 953 (10 878 185 to 11 797 812)	−1·5% (−1·9 to −1·1)	496 293 (322 502 to 691 283)	−0·4% (−1·0 to 0·2)	17 297 990 (15 567 817 to 19 198 683)	−5·5% (−6·2 to −4·7)	77 508 (49 695 to 113 154)	0·1% (−1·8 to 1·7)
**Southern Latin America**	**10 694 738 (10 201 211 to 11 200 269)**	**−1·5% (−3·1 to 0·2)**	**445 462 (284 632 to 625 408)**	**−0·7% (−3·0 to 1·8)**	**17 026 105 (15 295 127 to 18 926 015)**	**−3·0% (−5·4 to −0·5)**	**62 042 (39 481 to 90 851)**	**0·8% (−2·5 to 4·7)**
Argentina	7 110 301 (6 764 619 to 7 449 734)	−1·2% (−3·4 to 1·2)	295 691 (188 860 to 414 845)	−0·5% (−3·8 to 2·8)	11 317 606 (10 167 144 to 12 619 260)	−3·6% (−6·6 to −0·4)	40 891 (26 069 to 60 175)	0·4% (−4·4 to 5·5)
Chile	3 026 313 (2 883 224 to 3 173 943)	−2·1% (−4·4 to 0·3)	126 395 (80 166 to 177 701)	−0·9% (−4·2 to 2·5)	4 807 703 (4 316 782 to 5 321 021)	−1·7% (−5·5 to 2·9)	17 842 (11 347 to 26 106)	1·7% (−3·2 to 7·0)
Uruguay	557 656 (528 219 to 588 574)	−1·7% (−4·2 to 0·8)	23 356 (15 013 to 32 994)	−1·0% (−4·3 to 2·5)	900 049 (808 032 to 997 326)	−3·2% (−5·9 to −0·1)	3306 (2098 to 4868)	0·3% (−4·7 to 5·7)
**Eastern Europe**	**36 522 399 (34 831 810 to 38 180 675)**	**−1·1% (−2·6 to 0·3)**	**1 666 165 (1 087 007 to 2 308 943)**	**0·0% (−2·4 to 2·3)**	**60 047 323 (54 252 621 to 66 001 997)**	**−6·4% (−9·6 to −3·4)**	**289 888 (183 340 to 429 480)**	**−0·4% (−4·1 to 3·9)**
Belarus	1 699 640 (1 593 984 to 1 807 785)	−1·3% (−4·0 to 1·1)	78 840 (50 929 to 109 943)	−0·1% (−3·7 to 3·5)	2 902 221 (2 611 148 to 3 211 430)	−4·1% (−7·0 to −0·9)	14 267 (8699 to 21 853)	0·6% (−5·2 to 6·5)
Estonia	225 237 (211 324 to 239 545)	−2·2% (−4·8 to 0·5)	10 576 (6831 to 14 772)	−0·5% (−4·4 to 3·6)	382 224 (341 190 to 422 705)	−4·2% (−7·2 to −0·9)	1951 (1206 to 3012)	0·7% (−4·9 to 7·3)
Latvia	342 799 (321 121 to 365 126)	−1·7% (−4·5 to 0·9)	16 031 (10 423 to 22 579)	−0·1% (−3·8 to 4·0)	583 339 (522 934 to 646 660)	−3·3% (−6·3 to −0·2)	2947 (1807 to 4490)	1·1% (−4·6 to 7·5)
Lithuania	510 354 (487 671 to 532 005)	−1·3% (−3·5 to 0·7)	22 244 (14 485 to 31 106)	−0·2% (−3·5 to 3·0)	903 696 (812 361 to 1 000 663)	−2·4% (−5·0 to 0·4)	3679 (2366 to 5314)	0·5% (−4·0 to 5·5)
Moldova	745 403 (698 236 to 792 635)	−1·7% (−4·1 to 0·8)	34 136 (22 171 to 47 267)	−0·4% (−3·7 to 3·1)	1 337 141 (1 191 886 to 1 488 115)	−3·1% (−5·9 to −0·2)	6084 (3702 to 9254)	0·6% (−4·9 to 6·6)
Russia	24 812 212 (23 707 438 to 25 854 358)	−0·9% (−2·9 to 1·0)	1 124 516 (730 163 to 1 560 488)	0·2% (−2·8 to 3·3)	39 795 927 (36 096 715 to 43 757 724)	−7·9% (−12·6 to −3·5)	191 895 (122 723 to 281 246)	−0·8% (−6·0 to 4·4)
Ukraine	8 186 753 (7 663 105 to 8 701 072)	−1·2% (−3·9 to 1·4)	379 822 (245 301 to 529 894)	−0·1% (−3·9 to 4·0)	14 142 775 (12 662 586 to 15 739 619)	−2·5% (−5·4 to 0·4)	69 066 (41 700 to 106 641)	1·0% (−4·6 to 7·1)
**Central Europe**	**18 993 317 (17 924 101 to 20 107 210)**	**−1·4% (−2·4 to −0·4)**	**895 292 (580 928 to 1 249 464)**	**−0·4% (−1·7 to 1·1)**	**29 140 306 (26 070 435 to 32 262 618)**	**−5·3% (−6·5 to −4·0)**	**160 015 (100 777 to 236 592)**	**−0·4% (−2·7 to 2·2)**
Albania	470 177 (443 428 to 497 241)	0·1% (−2·2 to 2·7)	21 744 (13 898 to 30 469)	1·1% (−2·3 to 4·5)	751 910 (672 406 to 842 094)	−5·1% (−7·8 to −1·9)	3741 (2351 to 5531)	0·8% (−4·0 to 6·0)
Bosnia and Herzegovina	639 461 (600 415 to 677 877)	−2·2% (−4·5 to 0·2)	29 735 (19 404 to 41 666)	−0·9% (−4·1 to 2·5)	1 013 644 (904 473 to 1 132 866)	−8·0% (−11·1 to −4·8)	5267 (3305 to 7790)	−0·3% (−5·5 to 5·0)
Bulgaria	1 181 688 (1 108 013 to 1 252 908)	−1·5% (−3·9 to 1·0)	56 032 (36 529 to 78 549)	−0·7% (−4·2 to 2·8)	1 815 845 (1 625 273 to 2 016 449)	−4·4% (−7·4 to −1·3)	10 155 (6391 to 14 963)	−0·7% (−5·8 to 4·8)
Croatia	679 780 (651 507 to 708 132)	−1·0% (−2·8 to 0·9)	32 184 (20 986 to 44 579)	−0·4% (−3·5 to 2·8)	1 033 878 (929 465 to 1 144 445)	−4·6% (−8·1 to −1·4)	5775 (3674 to 8531)	−1·1% (−5·7 to 4·4)
Czech Republic	1 722 598 (1 621 742 to 1 823 581)	−1·4% (−4·2 to 1·3)	81 407 (52 955 to 114 677)	−0·9% (−4·4 to 2·5)	2 614 501 (2 338 857 to 2 906 582)	−4·1% (−7·1 to −1·0)	14 690 (9256 to 21 746)	−1·1% (−5·7 to 4·2)
Hungary	1 629 447 (1 531 918 to 1 723 579)	−1·3% (−3·6 to 1·1)	76 879 (50 433 to 107 353)	−0·3% (−3·9 to 3·2)	2 492 086 (2 243 767 to 2 761 247)	−4·8% (−7·8 to −2·0)	13 787 (8661 to 20 435)	−0·2% (−5·2 to 5·3)
Macedonia	345 962 (326 144 to 366 814)	−1·0% (−3·4 to 1·6)	16 170 (10 486 to 22 701)	−0·3% (−3·7 to 3·1)	539 461 (484 933 to 602 506)	−4·3% (−7·3 to −1·2)	2832 (1782 to 4214)	−0·4% (−5·2 to 4·8)
Montenegro	101 028 (95 248 to 106 640)	−0·8% (−3·3 to 1·8)	4722 (3068 to 6584)	−0·6% (−3·8 to 2·7)	156 302 (139 761 to 173 969)	−3·2% (−6·1 to −0·2)	830 (523 to 1219)	−0·8% (−5·7 to 4·9)
Poland	6 377 038 (6 009 928 to 6 774 657)	−1·7% (−3·9 to 0·7)	301 540 (195 947 to 422 082)	−0·3% (−3·4 to 3·1)	9 750 850 (8 677 425 to 10 895 665)	−6·3% (−9·3 to −3·2)	53 962 (33 946 to 79 509)	−0·1% (−5·2 to 5·7)
Romania	3 174 876 (2 988 054 to 3 362 001)	−1·1% (−3·3 to 1·3)	149 483 (97 035 to 207 629)	0·0% (−3·2 to 3·3)	4 843 083 (4 363 104 to 5 372 688)	−5·0% (−7·8 to −2·0)	26 671 (16 888 to 39 894)	−0·2% (−5·0 to 5·6)
Serbia	1 430 906 (1 351 907 to 1 517 239)	−0·9% (−3·1 to 1·5)	66 755 (43 486 to 92 732)	−0·5% (−3·8 to 2·9)	2 246 471 (2 015 088 to 2 496 754)	−3·6% (−6·4 to −0·3)	11 808 (7429 to 17 420)	−0·8% (−5·5 to 4·5)
Slovakia	908 113 (855 264 to 963 273)	−1·5% (−3·9 to 1·0)	42 923 (27 978 to 60 511)	−0·6% (−3·9 to 3·0)	1 380 345 (1 231 379 to 1 537 857)	−4·7% (−7·5 to −1·8)	7656 (4827 to 11 471)	−0·6% (−5·4 to 4·6)
Slovenia	332 244 (313 442 to 351 811)	−1·7% (−3·9 to 0·5)	15 718 (10 299 to 21 991)	−1·1% (−4·2 to 2·2)	501 929 (449 749 to 558 381)	−4·3% (−7·2 to −1·2)	2842 (1807 to 4202)	−1·3% (−5·7 to 3·6)
**Central Asia**	**13 736 082 (12 923 508 to 14 552 995)**	**−1·2% (−2·4 to 0·0)**	**590 288 (376 648 to 822 544)**	**−0·3% (−1·9 to 1·3)**	**24 381 639 (21 748 430 to 27 281 199)**	**−4·2% (−5·5 to −2·8)**	**91 106 (57 961 to 134 027)**	**−0·3% (−2·6 to 2·0)**
Armenia	504 582 (474 328 to 536 134)	−0·1% (−2·6 to 2·5)	21 922 (14 029 to 30 499)	0·8% (−2·8 to 4·3)	872 662 (778 002 to 971 362)	−4·2% (−7·1 to −1·0)	3459 (2197 to 5037)	0·2% (−4·2 to 5·5)
Azerbaijan	1 600 921 (1 502 383 to 1 704 523)	−1·9% (−4·6 to 0·7)	69 408 (44 683 to 96 691)	−1·0% (−4·2 to 2·6)	2 774 204 (2 459 259 to 3 130 530)	−4·8% (−7·8 to −1·7)	10 807 (6885 to 15 956)	−0·8% (−5·3 to 3·8)
Georgia	647 211 (607 406 to 688 166)	−1·1% (−3·5 to 1·5)	28 229 (18 220 to 39 473)	−0·5% (−3·7 to 3·0)	1 123 285 (1 004 197 to 1 246 394)	−2·3% (−5·0 to 0·6)	4510 (2862 to 6646)	−0·2% (−4·6 to 5·1)
Kazakhstan	2 766 716 (2 596 630 to 2 930 998)	−1·0% (−3·6 to 1·8)	119 073 (75 684 to 165 930)	−0·2% (−3·5 to 3·3)	4 822 123 (4 316 229 to 5 383 920)	−3·5% (−6·5 to −0·3)	18 530 (11 814 to 27 281)	0·0% (−4·7 to 5·3)
Kyrgyzstan	901 584 (846 088 to 954 712)	−0·7% (−3·4 to 2·0)	38 461 (24 434 to 54 387)	−0·1% (−3·4 to 3·4)	1 645 510 (1 470 878 to 1 835 833)	−1·9% (−4·7 to 1·2)	5912 (3703 to 8746)	0·3% (−4·1 to 5·8)
Mongolia	472 326 (442 558 to 502 979)	−1·3% (−3·8 to 1·3)	20 224 (12 875 to 28 503)	−0·5% (−3·9 to 3·2)	845 589 (751 829 to 946 192)	−5·1% (−7·9 to −2·3)	3104 (1957 to 4517)	−0·7% (−5·2 to 4·6)
Tajikistan	1 262 201 (1 184 935 to 1 339 385)	−1·2% (−3·7 to 1·6)	53 706 (34 428 to 75 752)	−0·4% (−3·6 to 3·1)	2 359 192 (2 092 141 to 2 630 580)	−3·0% (−5·7 to 0·1)	8165 (5188 to 12 137)	−0·2% (−5·1 to 5·0)
Turkmenistan	855 817 (801 642 to 908 000)	−1·9% (−4·5 to 0·7)	36 916 (23 531 to 51 851)	−0·4% (−3·6 to 3·1)	1 502 813 (1 337 795 to 1 688 181)	−7·3% (−10·4 to −4·3)	5635 (3576 to 8275)	−0·7% (−5·7 to 4·8)
Uzbekistan	4 724 725 (4 438 003 to 5 015 881)	−1·4% (−4·2 to 1·3)	202 349 (129 874 to 280 721)	−0·1% (−3·6 to 3·4)	8 436 261 (7 474 208 to 9 462 315)	−6·3% (−9·1 to −3·4)	30 984 (19 569 to 45 265)	−0·5% (−5·3 to 4·8)
**Central Latin America**	**34 762 545 (33 133 735 to 36 289 519)**	**−1·9% (−2·5 to −1·2)**	**1 660 602 (1 076 145 to 2 304 492)**	**0·1% (−0·9 to 1·3)**	**72 991 274 (65 889 598 to 81 184 037)**	**−8·9% (−9·9 to −7·9)**	**322 845 (208 259 to 471 825)**	**−0·1% (−2·8 to 2·6)**
Colombia	7 037 533 (6 687 851 to 7 402 769)	−1·9% (−4·1 to 0·3)	336 947 (217 516 to 466 079)	0·1% (−3·1 to 3·5)	13 988 578 (12 563 213 to 15 554 198)	−11·8% (−14·4 to −9·0)	64 386 (41 256 to 95 361)	−1·1% (−6·2 to 4·5)
Costa Rica	690 578 (650 177 to 731 940)	−1·6% (−4·1 to 0·9)	33 305 (21 674 to 46 323)	0·2% (−3·2 to 3·6)	1 386 207 (1 245 777 to 1 541 792)	−11·7% (−14·5 to −9·1)	6461 (4131 to 9495)	−1·2% (−6·4 to 4·8)
El Salvador	861 889 (810 452 to 915 206)	−0·6% (−3·1 to 2·1)	40 795 (26 577 to 57 355)	1·5% (−1·9 to 4·9)	1 782 909 (1 604 549 to 1 991 133)	−12·2% (−14·9 to −9·4)	7794 (4950 to 11 433)	−0·6% (−5·7 to 5·3)
Guatemala	2 118 201 (1 984 884 to 2 241 738)	−1·3% (−3·5 to 1·2)	98 348 (63 165 to 136 729)	1·1% (−2·4 to 4·6)	4 583 592 (4 085 517 to 5 140 291)	−12·0% (−14·7 to −8·9)	18 534 (11 815 to 27 162)	−0·6% (−6·0 to 5·2)
Honduras	1 099 359 (1 029 263 to 1 165 020)	−1·8% (−4·3 to 0·8)	51 332 (32 866 to 72 093)	0·2% (−3·3 to 3·9)	2 345 459 (2 102 884 to 2 628 176)	−12·1% (−14·8 to −9·2)	9695 (6194 to 14 381)	−1·3% (−6·1 to 4·1)
Mexico	17 294 301 (16 556 972 to 18 007 210)	−2·2% (−2·6 to −1·7)	829 440 (540 632 to 1 149 201)	−0·1% (−1·0 to 0·9)	37 432 220 (33 861 880 to 41 615 456)	−6·2% (−7·2 to −5·1)	164 143 (105 453 to 238 876)	0·9% (−1·4 to 2·9)
Nicaragua	843 871 (793 925 to 895 439)	−1·6% (−4·1 to 0·9)	39 737 (25 685 to 55 728)	0·2% (−3·2 to 3·9)	1 768 388 (1 582 359 to 1 971 937)	−11·4% (−14·1 to −8·6)	7517 (4844 to 11 172)	−1·5% (−6·6 to 4·4)
Panama	540 954 (507 859 to 573 257)	−1·5% (−4·0 to 1·1)	26 001 (16 679 to 36 516)	0·2% (−3·0 to 3·9)	1 083 812 (969 998 to 1 210 006)	−11·4% (−14·0 to −8·5)	5021 (3174 to 7420)	−1·1% (−6·0 to 4·7)
Venezuela	4 275 858 (4 072 177 to 4 503 332)	−1·3% (−3·7 to 1·1)	204 698 (132 318 to 283 996)	0·5% (−2·8 to 4·1)	8 620 107 (7 746 111 to 9 650 749)	−11·4% (−14·1 to −8·6)	39 293 (24 872 to 57 642)	−1·0% (−5·8 to 4·9)
**Andean Latin America**	**7 900 228 (7 520 345 to 8 280 881)**	**−2·1% (−3·6 to −0·7)**	**369 316 (238 655 to 519 276)**	**−0·5% (−2·5 to 1·9)**	**15 134 012 (13 576 568 to 16 934 973)**	**−8·5% (−10·5 to −6·4)**	**67 651 (42 518 to 100 250)**	**−0·8% (−4·3 to 3·0)**
Bolivia	1 384 973 (1 301 307 to 1 468 594)	−2·1% (−4·6 to 0·5)	64 043 (41 372 to 89 926)	0·0% (−3·6 to 3·8)	2 772 129 (2 483 303 to 3 114 809)	−10·7% (−13·7 to −7·9)	11 777 (7 412 to 17 352)	−1·1% (−6·2 to 4·7)
Ecuador	2 045 883 (1 943 350 to 2 147 691)	−1·6% (−3·8 to 0·6)	97 541 (62 821 to 137 300)	0·0% (−3·4 to 3·1)	4 184 493 (3 746 562 to 4 690 628)	−9·3% (−12·1 to −6·4)	18 790 (11 909 to 27 908)	−1·1% (−5·9 to 4·4)
Peru	4 469 372 (4 253 912 to 4 684 114)	−2·1% (−4·1 to 0·0)	207 731 (133 541 to 292 162)	−0·6% (−3·7 to 2·9)	8 177 390 (7 313 037 to 9 128 152)	−7·5% (−10·5 to −4·1)	37 084 (23 233 to 55 383)	−0·6% (−5·6 to 4·7)
**Caribbean**	**6 508 546 (6 133 601 to 6 894 055)**	**−1·3% (−2·5 to −0·2)**	**302 360 (195 258 to 420 752)**	**−0·3% (−1·9 to 1·4)**	**13 706 101 (12 409 230 to 15 187 209)**	**−8·0% (−9·2 to −6·6)**	**57 484 (36 307 to 85 384)**	**−1·8% (−4·5 to 0·9)**
Antigua and Barbuda	13 734 (12 885 to 14 543)	−0·6% (−3·1 to 2·0)	643 (413 to 896)	0·1% (−3·2 to 3·5)	27 539 (24 789 to 30 742)	−8·0% (−10·5 to −5·1)	121 (76 to 179)	−1·7% (−6·4 to 3·1)
The Bahamas	58 695 (55 343 to 62 407)	−1·3% (−3·8 to 1·5)	2762 (1787 to 3880)	−0·7% (−3·9 to 2·7)	117 526 (105 569 to 131 008)	−7·4% (−10·0 to −4·5)	526 (332 to 776)	−2·1% (−6·7 to 2·7)
Barbados	41 798 (39 329 to 44 221)	−1·5% (−3·8 to 1·0)	1987 (1298 to 2775)	−0·9% (−4·4 to 2·7)	84 944 (76 343 to 94 328)	−7·6% (−10·4 to −4·8)	389 (245 to 570)	−2·3% (−6·9 to 2·6)
Belize	50 816 (47 780 to 54 077)	−1·2% (−3·6 to 1·5)	2316 (1484 to 3290)	0·1% (−3·2 to 3·5)	108 679 (97 158 to 121 977)	−10·1% (−12·8 to −7·1)	425 (268 to 624)	−1·7% (−6·4 to 3·8)
Bermuda	10 187 (9558 to 10 788)	−1·2% (−3·8 to 1·3)	481 (312 to 675)	−0·1% (−3·5 to 3·4)	20 501 (18 500 to 22 854)	−8·0% (−10·5 to −5·2)	91 (57 to 136)	−1·7% (−6·4 to 3·2)
Cuba	1 708 831 (1 601 812 to 1 816 682)	−1·6% (−3·9 to 0·9)	81 684 (53 101 to 114 169)	−0·3% (−3·6 to 2·9)	3 454 818 (3 109 983 to 3 847 980)	−8·3% (−10·8 to −5·5)	15 995 (10 081 to 23 690)	−1·7% (−6·2 to 3·4)
Dominica	10 714 (10 066 to 11 357)	−1·7% (−4·1 to 0·8)	501 (323 to 700)	−0·4% (−3·9 to 3·1)	22 146 (19 928 to 24 630)	−9·7% (−12·4 to −6·9)	95 (59 to 139)	−1·9% (−6·5 to 3·7)
Dominican Republic	1 469 656 (1 382 391 to 1 552 206)	−1·2% (−3·8 to 1·4)	68 151 (43 787 to 94 794)	0·1% (−3·1 to 3·5)	3 092 021 (2 786 604 to 3 444 270)	−10·1% (−12·4 to −7·1)	12 760 (8145 to 19 063)	−1·4% (−6·3 to 4·0)
Grenada	15 150 (14 259 to 16 091)	−3·1% (−5·6 to −0·6)	700 (452 to 980)	−0·9% (−4·2 to 2·5)	31 508 (28 222 to 35 245)	−12·2% (−14·8 to −9·6)	131 (84 to 193)	−2·2% (−6·9 to 3·6)
Guyana	108 275 (101 622 to 114 809)	−2·6% (−5·2 to −0·2)	4950 (3181 to 6960)	−1·4% (−4·8 to 2·1)	233 389 (209 966 to 262 659)	−9·7% (−12·5 to −6·9)	933 (585 to 1387)	−2·5% (−7·1 to 2·6)
Haiti	1 491 507 (1 395 490 to 1 583 879)	−1·9% (−4·5 to 0·7)	66 275 (42 627 to 92 230)	−0·4% (−3·9 to 3·1)	3 393 664 (3 058 577 to 3 768 652)	−8·7% (−11·2 to −6·1)	12 197 (7695 to 17 990)	−1·8% (−6·4 to 3·4)
Jamaica	406 638 (381 821 to 431 984)	−1·7% (−4·1 to 0·7)	18 859 (12 108 to 26 453)	−0·9% (−4·1 to 2·5)	848 127 (762 215 to 942 951)	−8·5% (−11·1 to −5·8)	3564 (2234 to 5258)	−2·4% (−6·8 to 3·1)
Puerto Rico	538 458 (506 854 to 571 270)	−2·0% (−4·3 to 0·7)	25 726 (16 718 to 36 404)	−0·9% (−4·3 to 2·6)	1 063 365 (955 245 to 1 186 178)	−9·2% (−12·0 to −6·4)	5005 (3144 to 7421)	−2·1% (−6·9 to 3·0)
Saint Lucia	27 432 (25 766 to 29 053)	−1·7% (−4·2 to 0·9)	1278 (821 to 1790)	0·3% (−3·3 to 3·8)	56 565 (50 863 to 62 888)	−10·2% (−12·7 to −7·3)	242 (152 to 362)	−1·3% (−6·2 to 4·1)
Saint Vincent and the Grenadines	15 937 (14 982 to 16 898)	−2·5% (−4·9 to 0·2)	738 (476 to 1029)	−1·2% (−4·4 to 2·4)	33 500 (30 097 to 37 170)	−10·3% (−13·0 to −7·3)	140 (89 to 209)	−2·5% (−7·1 to 2·8)
Suriname	78 191 (73 764 to 82 955)	−1·3% (−3·8 to 1·0)	3616 (2353 to 5093)	−0·5% (−4·1 to 2·8)	162 947 (146 639 to 182 079)	−9·2% (−12·1 to −6·6)	682 (428 to 1007)	−2·0% (−6·6 to 3·2)
Trinidad and Tobago	204 165 (191 574 to 216 194)	−1·5% (−4·1 to 0·9)	9576 (6159 to 13 407)	−0·5% (−3·9 to 2·8)	412 057 (369 602 to 457 568)	−8·5% (−11·4 to −5·6)	1833 (1166 to 2720)	−2·0% (−6·4 to 3·4)
Virgin Islands	14 900 (14 018 to 15 792)	−1·1% (−3·5 to 1·4)	716 (467 to 1 004)	−0·4% (−3·9 to 2·8)	30 156 (27 395 to 33 517)	−8·3% (−11·0 to −5·7)	142 (91 to 211)	−1·9% (−6·4 to 3·5)
**Tropical Latin America**	**34 400 664 (32 950 311 to 35 875 803)**	**−2·3% (−3·0 to −1·7)**	**1 509 125 (969 280 to 2 104 691)**	**−0·7% (−1·8 to 0·4)**	**79 012 630 (71 914 512 to 86 871 666)**	**−9·4% (−10·4 to −8·5)**	**272 278 (173 288 to 400 837)**	**−2·1% (−4·7 to 0·9)**
Brazil	33 392 533 (31 997 019 to 34 814 190)	−2·3% (−3·0 to −1·7)	1 465 378 (941 229 to 2 043 622)	−0·7% (−1·8 to 0·4)	76 805 591 (69 918 194 to 84 431 513)	−9·4% (−10·4 to −8·4)	264 748 (168 571 to 389 437)	−2·0% (−4·7 to 0·9)
Paraguay	1 008 131 (952 308 to 1 067 392)	−2·6% (−5·2 to −0·1)	43 747 (27 869 to 61 297)	−1·3% (−4·7 to 2·1)	2 207 039 (1 988 371 to 2 456 628)	−11·5% (−13·9 to −9·1)	7530 (4730 to 11 145)	−3·0% (−7·7 to 2·6)
**East Asia**	**138 171 702 (132 478 067 to 143 745 425)**	**−1·9% (−2·4 to −1·4)**	**6 165 860 (4 001 597 to 8 612 689)**	**−0·2% (−1·2 to 0·8)**	**269 879 994 (239 778 320 to 303 834 702)**	**−10·9% (−11·9 to −10·0)**	**1 058 408 (673 059 to 1 557 133)**	**−2·1% (−5·0 to 0·9)**
China	132 930 661 (127 445 922 to 138 273 863)	−1·9% (−2·4 to −1·4)	5 938 315 (3 853 623 to 8 301 696)	−0·2% (−1·2 to 0·9)	260 794 517 (231 640 907 to 293 800 999)	−11·2% (−12·2 to −10·2)	1 022 673 (649 953 to 1 503 383)	−2·2% (−5·2 to 0·9)
North Korea	2 593 897 (2 448 706 to 2 746 024)	−1·8% (−4·5 to 0·7)	112 513 (71 390 to 159 152)	−1·8% (−5·6 to 2·0)	4 963 157 (4 412 118 to 5 605 148)	0·0% (−3·5 to 3·8)	18 288 (11 376 to 27 487)	−1·3% (−6·9 to 4·0)
Taiwan (province of China)	2 647 143 (2530 864 to 2 761 814)	−0·5% (−2·5 to 1·7)	115 031 (73 874 to 160 100)	1·2% (−2·7 to 5·5)	4 122 320 (3 650 654 to 4 662 418)	−4·9% (−7·9 to −2·1)	17 447 (11 118 to 25 979)	2·0% (−3·5 to 8·3)
**Southeast Asia**	**84 397 472 (80 130 911 to 88 755 912)**	**−2·4% (−3·1 to −1·6)**	**3 466 178 (2 197 749 to 4 878 575)**	**−1·0% (−2·1 to 0·2)**	**176 724 151 (157 991 896 to 197 116 698)**	**−6·7% (−7·6 to −5·8)**	**524 458 (325 896 to 794 140)**	**−1·4% (−3·6 to 1·3)**
Cambodia	1 972 188 (1 850 593 to 2 098 567)	−2·8% (−5·5 to −0·3)	79 763 (50 572 to 113 503)	−0·5% (−4·4 to 3·4)	4 282 356 (3 807 064 to 4 787 193)	−7·6% (−10·2 to −4·8)	11 963 (7381 to 18 296)	−1·3% (−6·1 to 4·1)
Indonesia	33 130 967 (31 667 129 to 34 597 261)	−2·4% (−3·2 to −1·7)	1 359 211 (865 077 to 1 909 535)	−1·3% (−2·5 to 0·0)	71 377 953 (63 879 623 to 79 957 483)	−6·4% (−7·3 to −5·3)	208 622 (129 418 to 317 615)	−1·5% (−3·7 to 1·2)
Laos	819 097 (768 588 to 871 911)	−2·3% (−4·8 to 0·4)	33 283 (20 889 to 47 575)	−0·3% (−4·3 to 3·4)	1 803 437 (1 602 489 to 2 042 738)	−7·4% (−10·1 to −4·5)	4992 (3066 to 7670)	−1·5% (−6·1 to 4·6)
Malaysia	3 911 967 (3 686 622 to 4 142 386)	−1·8% (−4·2 to 0·7)	161 953 (102 972 to 228 271)	−0·6% (−4·2 to 3·3)	7 989 485 (7 067 898 to 9 022 928)	−7·6% (−10·5 to −4·6)	24 399 (15 173 to 36 772)	−1·6% (−6·5 to 4·3)
Maldives	47 268 (44 250 to 50 146)	−1·0% (−3·6 to 1·9)	1935 (1232 to 2744)	1·1% (−2·8 to 5·2)	97 407 (85 814 to 109 722)	−8·3% (−11·0 to −5·5)	286 (178 to 436)	−0·9% (−6·1 to 5·7)
Mauritius	171 325 (160 870 to 182 098)	−1·9% (−4·4 to 0·7)	7042 (4454 to 9993)	−0·9% (−4·5 to 2·7)	339 204 (304 007 to 380 540)	−5·7% (−8·7 to −2·7)	1066 (671 to 1594)	−0·9% (−5·9 to 5·0)
Myanmar	7 065 448 (6 641 084 to 7 496 340)	−2·7% (−5·3 to 0·0)	288 950 (184 100 to 409 943)	−0·5% (−4·2 to 3·2)	14 831 091 (13 211 092 to 16 598 830)	−8·8% (−11·5 to −5·8)	43 369 (26 887 to 65 583)	−2·0% (−7·1 to 4·2)
Philippines	12 301 469 (11 519 365 to 13 053 083)	−1·6% (−4·0 to 0·8)	503 476 (319 478 to 716 513)	−0·3% (−3·9 to 3·4)	25 812 371 (22 895 135 to 29 075 489)	−4·6% (−7·4 to −1·5)	75 184 (46 313 to 114 014)	−0·4% (−5·5 to 4·8)
Sri Lanka	2 685 239 (2 516 271 to 2 854 287)	−0·5% (−3·1 to 2·0)	110 736 (70 439 to 157 347)	0·8% (−3·1 to 4·9)	5 407 181 (4 836 171 to 6 038 976)	−6·0% (−9·1 to −2·8)	16 703 (10 412 to 25 103)	−0·2% (−5·3 to 5·3)
Seychelles	12 522 (11 746 to 13 303)	−2·4% (−4·9 to 0·1)	518 (328 to 735)	−1·4% (−5·5 to 2·6)	25 010 (22 211 to 28 102)	−5·6% (−8·5 to −2·5)	78 (49 to 117)	−1·2% (−5·8 to 4·5)
Thailand	9 517 252 (8 993 897 to 10 052 983)	−2·6% (−4·9 to −0·3)	393 165 (252 248 to 555 245)	−1·2% (−5·1 to 2·4)	18 391 437 (16 475 970 to 20 522 514)	−7·1% (−10·1 to −4·3)	58 561 (36 905 to 87 121)	−1·5% (−6·2 to 4·2)
Timor-Leste	124 351 (116 464 to 132 519)	−2·6% (−5·2 to 0·0)	5038 (3202 to 7140)	−0·7% (−4·5 to 2·9)	274 567 (244 943 to 310 145)	−7·9% (−10·8 to −4·9)	759 (463 to 1169)	−1·7% (−6·4 to 4·1)
Vietnam	12 526 388 (11 733 212 to 13 291 801)	−2·8% (−5·5 to 0·0)	516 501 (328 938 to 728 504)	−1·2% (−4·9 to 2·5)	25 861 953 (22 892 742 to 28 991 367)	−7·8% (−10·4 to −4·8)	77 772 (48 261 to 117 920)	−1·8% (−6·9 to 4·1)
**Oceania**	**1 160 780 (1 092 102 to 1 231 905)**	**−1·3% (−3·2 to 0·6)**	**47 331 (29 927 to 66 877)**	**−0·7% (−3·6 to 2·0)**	**2 498 945 (2 220 061 to 2 820 943)**	**−4·2% (−6·2 to −1·9)**	**7356 (4600 to 11 094)**	**−0·9% (−4·4 to 2·9)**
American Samoa	8396 (7890 to 8920)	−0·4% (−3·0 to 2·3)	347 (222 to 494)	0·1% (−3·6 to 4·1)	16 352 (14 407 to 18 430)	−4·4% (−7·7 to −1·2)	53 (33 to 79)	−0·3% (−5·4 to 5·5)
Federated States of Micronesia	10 657 (9996 to 11 313)	−2·3% (−4·8 to 0·2)	438 (277 to 624)	−1·4% (−5·0 to 2·6)	22 231 (19 603 to 25 296)	−7·2% (−9·9 to −4·1)	67 (42 to 101)	−1·9% (−6·9 to 3·7)
Fiji	97 017 (91 474 to 102 778)	−2·0% (−4·5 to 0·5)	4015 (2549 to 5644)	−1·1% (−5·0 to 2·9)	189 834 (167 528 to 214 803)	−5·6% (−8·5 to −2·4)	616 (388 to 912)	−1·1% (−6·0 to 4·3)
Guam	18 623 (17 552 to 19 718)	0·9% (−1·7 to 3·3)	781 (504 to 1101)	1·0% (−2·9 to 4·9)	35 267 (31 369 to 39 835)	−1·4% (−4·4 to 1·7)	120 (77 to 177)	0·7% (−4·8 to 6·5)
Kiribati	12 021 (11 270 to 12 733)	−1·6% (−4·2 to 1·3)	490 (311 to 688)	−0·9% (−4·9 to 2·9)	25 841 (22 940 to 29 294)	−5·3% (−8·3 to −2·0)	76 (47 to 114)	−1·3% (−6·3 to 4·0)
Marshall Islands	7520 (7081 to 7958)	−1·3% (−3·8 to 1·6)	307 (195 to 435)	−1·1% (−5·0 to 2·9)	15 690 (13 851 to 17 837)	−4·4% (−7·6 to −1·1)	47 (30 to 71)	−1·4% (−6·5 to 4·0)
Northern Mariana Islands	15 147 (14 178 to 16 163)	1·2% (−1·5 to 3·8)	630 (401 to 889)	1·5% (−2·5 to 5·5)	29 416 (25 480 to 34 090)	−2·4% (−5·5 to 0·9)	93 (58 to 142)	0·5% (−4·7 to 6·5)
Papua New Guinea	800 421 (752 037 to 850 011)	−1·5% (−4·2 to 0·9)	32 504 (20 454 to 45 996)	−0·9% (−4·7 to 2·9)	1 757 374 (1 559 341 to 1 979 788)	−4·7% (−7·4 to −1·7)	5063 (3164 to 7690)	−1·2% (−5·9 to 3·9)
Samoa	18 992 (17 885 to 20 142)	−2·4% (−5·0 to 0·0)	786 (497 to 1116)	−1·6% (−5·3 to 2·2)	39 840 (35 453 to 45 123)	−5·0% (−8·2 to −2·0)	122 (77 to 182)	−1·5% (−6·5 to 4·2)
Solomon Islands	59 294 (55 709 to 62 863)	−1·0% (−3·5 to 1·4)	2 418 (1 535 to 3 425)	−0·5% (−4·2 to 3·6)	129 342 (114 221 to 146 662)	−4·6% (−7·3 to −1·7)	374 (231 to 568)	−1·1% (−5·7 to 4·2)
Tonga	10 616 (9 977 to 11 202)	−1·9% (−4·2 to 0·8)	437 (282 to 615)	−1·1% (−4·6 to 2·8)	22 090 (19 660 to 25 069)	−5·3% (−8·4 to −2·1)	67 (42 to 101)	−1·2% (−6·2 to 4·9)
Vanuatu	27 789 (26 078 to 29 545)	−1·1% (−3·6 to 1·5)	1134 (716 to 1609)	−0·4% (−4·1 to 3·3)	59 608 (52 429 to 67 509)	−5·1% (−8·0 to −1·9)	175 (109 to 267)	−0·8% (−5·5 to 5·1)
**North Africa and Middle East**	**93 437 442 (88 329 677 to 98 428 935)**	**−2·3% (−3·1 to −1·5)**	**4 289 081 (2 774 191 to 5 940 727)**	**1·1% (−0·4 to 2·6)**	**175 091 388 (156 773 718 to 194 351 068)**	**−8·5% (−9·6 to −7·3)**	**777 533 (495 248 to 1 158 025)**	**3·2% (−0·4 to 6·7)**
Afghanistan	4 841 208 (4 533 619 to 5 141 654)	−1·9% (−4·5 to 0·9)	208 981 (134 134 to 294 141)	0·5% (−3·4 to 4·4)	9 939 058 (8 934 823 to 11 103 449)	−4·8% (−7·4 to −2·0)	35 909 (22 613 to 53 732)	1·5% (−3·1 to 6·5)
Algeria	6 651 343 (6 217 723 to 7 093 673)	−1·9% (−4·5 to 0·5)	309 251 (199 203 to 431 278)	1·2% (−2·2 to 4·3)	12 127 472 (10 825 364 to 13 521 074)	−7·9% (−10·6 to −5·1)	56 455 (35 743 to 84 961)	2·9% (−2·6 to 9·2)
Bahrain	240 046 (223 355 to 256 642)	−3·3% (−5·5 to −0·5)	11 300 (7222 to 15 841)	−0·6% (−4·2 to 3·2)	443 334 (390 844 to 499 432)	−7·0% (−10·2 to −4·0)	2112 (1327 to 3201)	1·4% (−4·1 to 6·8)
Egypt	14 623 084 (13 720 844 to 15 493 197)	−2·2% (−4·8 to 0·7)	674 197 (433 516 to 936 625)	0·9% (−2·5 to 4·9)	26 934 828 (24 165 685 to 30 075 283)	−7·3% (−10·0 to −4·6)	121 944 (77 062 to 181 851)	3·0% (−2·2 to 8·9)
Iran	13 616 955 (12 927 168 to 14 325 355)	−2·1% (−4·4 to −0·2)	653 679 (425 780 to 905 798)	2·3% (−1·0 to 5·7)	26 496 199 (23 627 819 to 29 527 389)	−12·3% (−15·6 to −9·1)	128 660 (82 720 to 188 190)	4·1% (−1·8 to 11·0)
Iraq	5 534 181 (5 162 542 to 5 886 763)	−0·9% (−3·4 to 1·7)	245 414 (157 277 to 342 808)	0·6% (−2·9 to 4·3)	10 732 501 (9 576 752 to 11 948 005)	−3·1% (−5·8 to −0·4)	42 924 (27 365 to 64 414)	1·7% (−3·1 to 7·1)
Jordan	1 203 358 (1 124 971 to 1 284 958)	−1·1% (−3·9 to 1·3)	55 454 (35 403 to 77 551)	1·9% (−1·7 to 5·5)	2 196 597 (1 951 993 to 2 462 165)	−7·2% (−10·1 to −4·7)	9934 (6276 to 14 876)	3·7% (−1·6 to 9·6)
Kuwait	712 246 (664 472 to 759 904)	−2·0% (−4·6 to 0·6)	33 859 (21 841 to 48 110)	0·6% (−3·1 to 4·3)	1 250 436 (1 101 203 to 1 414 499)	−7·2% (−9·9 to −4·4)	6281 (3929 to 9644)	2·6% (−2·9 to 8·9)
Lebanon	1 017 537 (954 285 to 1 084 062)	−2·8% (−5·5 to −0·1)	47 836 (30 941 to 66 597)	0·5% (−2·9 to 3·9)	1 795 208 (1 607 600 to 2 009 884)	−7·2% (−10·0 to −4·2)	8862 (5586 to 13 274)	2·8% (−2·3 to 8·7)
Libya	1 054 747 (988 490 to 1 121 886)	1·0% (−1·6 to 3·6)	49 500 (32 133 to 69 680)	3·3% (−0·3 to 7·3)	1 837 414 (1 632 454 to 2 055 257)	−6·2% (−8·9 to −3·5)	9053 (5661 to 13 629)	4·4% (−0·9 to 10·8)
Morocco	5 883 012 (5 509 597 to 6 260 127)	−2·1% (−4·6 to 0·5)	271 235 (174 130 to 382 333)	1·3% (−2·3 to 5·1)	10 851 241 (9 726 403 to 12 093 488)	−8·4% (−11·0 to −5·5)	49 517 (31 448 to 73 822)	3·3% (−2·3 to 9·4)
Oman	773 924 (720 555 to 827 174)	−9·8% (−12·1 to −7·3)	36 619 (23 376 to 50 763)	−3·5% (−7·4 to 0·9)	1 476 286 (1 287 874 to 1 688 025)	−19·2% (−21·9 to −16·4)	6862 (4261 to 10 363)	0·9% (−6·7 to 9·2)
Palestine	714 895 (666 691 to 761 373)	−0·6% (−3·0 to 1·9)	31 910 (20 345 to 44 967)	0·2% (−3·1 to 3·5)	1 399 904 (1 254 072 to 1 570 053)	−2·1% (−4·8 to 0·7)	5548 (3486 to 8251)	1·0% (−3·6 to 6·0)
Qatar	395 766 (367 481 to 425 083)	−3·3% (−6·0 to −0·8)	18 937 (12 171 to 26 563)	−0·8% (−4·3 to 3·1)	702 549 (610 417 to 805 880)	−5·4% (−8·6 to −2·2)	3540 (2191 to 5423)	2·1% (−3·5 to 8·2)
Saudi Arabia	5 797 325 (5 544 243 to 6 045 186)	−1·9% (−2·9 to −1·0)	267 107 (172 575 to 373 293)	2·8% (0·8 to 4·9)	8 683 665 (7 709 945 to 9 724 810)	−14·3% (−16·0 to −12·6)	44 858 (28 544 to 65 886)	5·3% (−0·3 to 10·5)
Sudan	6 119 599 (5 731 877 to 6 502 575)	−1·9% (−4·4 to 0·6)	273 586 (176 305 to 381 268)	1·2% (−2·2 to 4·7)	11 733 161 (10 534 241 to 13 096 213)	−7·8% (−10·4 to −5·0)	47 952 (30 469 to 71 699)	3·0% (−2·1 to 9·0)
Syria	2 864 674 (2 678 877 to 3 057 047)	−1·9% (−4·3 to 0·6)	130 923 (84 689 to 183 571)	1·4% (−2·0 to 5·3)	5 310 861 (4 745 133 to 5 968 312)	−8·9% (−11·7 to −6·1)	23 274 (14 638 to 34 911)	3·5% (−2·3 to 9·9)
Tunisia	1 898 520 (1 796 756 to 2 006 354)	−1·2% (−3·4 to 1·1)	90 158 (58 559 to 124 945)	1·8% (−1·5 to 5·4)	3 545 921 (3 184 205 to 3 944 544)	−7·9% (−10·5 to −5·1)	17 148 (10 721 to 25 799)	3·3% (−2·3 to 9·4)
Turkey	13 712 859 (13 037 034 to 14 355 744)	−2·4% (−4·7 to −0·1)	617 788 (397 735 to 854 395)	0·9% (−2·3 to 4·3)	26 323 266 (23 778 271 to 29 061 442)	−6·7% (−9·7 to −3·5)	109 395 (70 205 to 159 399)	3·6% (−1·6 to 9·5)
United Arab Emirates	1 566 357 (1 464 258 to 1 672 209)	−4·8% (−7·1 to −2·5)	76 336 (49 496 to 107 622)	−1·2% (−4·8 to 2·8)	3 050 507 (2 687 218 to 3 465 766)	−10·3% (−13·4 to −7·1)	15 103 (9215 to 23 288)	0·5% (−5·2 to 6·8)
Yemen	4 113 092 (3 847 322 to 4 385 146)	−3·9% (−6·1 to −1·6)	180 267 (115 752 to 254 144)	0·2% (−3·2 to 3·7)	8 070 505 (7 225 304 to 9 038 084)	−10·0% (−12·7 to −7·2)	31 337 (19 858 to 47 513)	2·5% (−2·9 to 9·2)
**South Asia**	**297 880 096 (285 188 495 to 310 043 695)**	**−2·6% (−3·1 to −2·1)**	**12 432 812 (7 979 873 to 17 372 734)**	**−0·6% (−1·5 to 0·4)**	**499 729 138 (450 049 525 to 558 211 468)**	**−14·1% (−15·3 to −12·8)**	**1 801 808 (1 142 781 to 2 654 364)**	**−2·6% (−6·2 to 1·2)**
Bangladesh	29 833 591 (28 066 427 to 31 671 803)	−2·3% (−4·9 to 0·2)	1 251 149 (799 359 to 1 755 583)	0·2% (−3·0 to 3·5)	49 777 226 (44 418 834 to 55 776 067)	−9·1% (−11·8 to −6·4)	180 304 (112 226 to 265 713)	0·4% (−4·6 to 6·3)
Bhutan	143 348 (134 491 to 151 726)	−4·4% (−6·7 to −1·9)	6028 (3879 to 8474)	−1·6% (−5·1 to 2·2)	237 971 (212 853 to 266 542)	−12·6% (−15·1 to −9·7)	879 (553 to 1295)	−0·7% (−6·4 to 6·1)
India	231 458 617 (221 593 942 to 240 869 588)	−2·7% (−3·2 to −2·3)	9 642 457 (6 188 454 to 13 483 361)	−0·7% (−1·6 to 0·1)	384 460 770 (346 179 104 to 429 907 693)	−15·3% (−16·7 to −13·9)	1 392 107 (883 830 to 2 038 166)	−3·4% (−7·2 to 0·6)
Nepal	5 671 848 (5 426 115 to 5 912 071)	−1·8% (−3·6 to 0·0)	234 171 (148 304 to 328 137)	0·5% (−2·4 to 3·5)	8 813 328 (7 993 756 to 9 847 113)	−5·1% (−7·9 to −2·1)	31 406 (19 939 to 46 563)	2·8% (−2·3 to 8·5)
Pakistan	30 772 692 (29 418 094 to 32 056 038)	−2·5% (−4·4 to −0·7)	1 299 007 (829 027 to 1 845 495)	−0·6% (−3·6 to 2·5)	56 439 842 (51 246 017 to 62 527 542)	−10·5% (−13·5 to −7·0)	197 112 (122 210 to 292 966)	−0·7% (−5·9 to 5·4)
**Southern sub-Saharan Africa**	**8 855 681 (8 430 740 to 9 247 481)**	**−1·2% (−2·0 to −0·4)**	**380 398 (245 291 to 530 950)**	**−1·0% (−2·2 to 0·3)**	**18 825 137 (16 695 800 to 21 235 581)**	**−4·8% (−5·9 to −3·8)**	**64 951 (41 251 to 95 803)**	**−0·7% (−2·8 to 1·6)**
Botswana	263 435 (248 179 to 279 636)	−2·9% (−5·5 to −0·2)	11 307 (7272 to 15 953)	−2·6% (−6·1 to 1·3)	535 013 (471 079 to 607 191)	−8·1% (−10·8 to −5·1)	1888 (1201 to 2812)	−2·5% (−7·4 to 3·1)
Lesotho	235 920 (221 739 to 250 093)	−3·3% (−5·7 to −0·6)	9931 (6307 to 13 930)	−3·4% (−6·9 to 0·2)	503 816 (444 480 to 572 909)	−7·8% (−10·7 to −4·8)	1655 (1042 to 2463)	−2·8% (−7·3 to 2·9)
Namibia	274 485 (257 443 to 290 730)	−2·1% (−4·5 to 0·5)	11 775 (7482 to 16 507)	−1·2% (−4·5 to 2·5)	568 190 (504 738 to 645 599)	−7·4% (−10·7 to −4·2)	1952 (1225 to 2915)	−1·2% (−6·5 to 4·6)
South Africa	6 276 496 (5 991 825 to 6 543 621)	−1·3% (−2·2 to −0·5)	270 768 (176 270 to 376 825)	−1·1% (−2·5 to 0·5)	13 305 448 (11 835 986 to 15 008 733)	−5·1% (−6·3 to −3·9)	46 821 (29 748 to 68 641)	−0·8% (−3·1 to 1·7)
Swaziland	140 193 (131 788 to 148 708)	−4·1% (−6·7 to −1·6)	5900 (3755 to 8360)	−4·7% (−8·3 to −0·9)	296 385 (260 404 to 338 444)	−7·0% (−9·8 to −3·7)	976 (621 to 1442)	−3·3% (−7·9 to 2·4)
Zimbabwe	1 665 152 (1 561 309 to 1 767 683)	−0·3% (−2·8 to 2·3)	70 716 (44 789 to 99 425)	−0·1% (−3·7 to 3·4)	3 616 284 (3 192 061 to 4 110 865)	−2·2% (−5·4 to 1·0)	11 658 (7328 to 17 455)	0·2% (−4·6 to 5·3)
**Western sub-Saharan Africa**	**43 730 076 (41 301 709 to 46 083 516)**	**−1·5% (−2·7 to −0·2)**	**1 812 846 (1 147 969 to 2 553 029)**	**0·2% (−1·7 to 2·2)**	**93 543 672 (83 595 903 to 104 836 362)**	**−2·5% (−3·9 to −1·1)**	**284 089 (177 657 to 427 908)**	**1·2% (−1·3 to 4·0)**
Benin	1 272 254 (1 204 978 to 1 339 904)	−3·1% (−5·5 to −0·9)	52 523 (33 155 to 74 425)	−0·9% (−4·3 to 2·7)	2 743 528 (2 442 961 to 3 091 386)	−3·8% (−6·8 to −0·9)	8212 (5110 to 12 303)	0·2% (−4·6 to 5·5)
Burkina Faso	2 043 309 (1 913 009 to 2 168 388)	−2·8% (−5·6 to −0·2)	83 979 (53 437 to 119 037)	0·0% (−3·8 to 3·9)	4 536 772 (4 040 129 to 5 083 218)	−3·6% (−6·2 to −0·8)	13 186 (8149 to 20 294)	1·0% (−3·4 to 6·2)
Cameroon	2 654 052 (2 490 969 to 2 813 638)	−1·6% (−4·5 to 1·0)	109 958 (69 463 to 155 810)	0·2% (−3·5 to 4·0)	5 632 723 (4 987 904 to 6 379 645)	−1·8% (−4·9 to 0·9)	17 156 (10 715 to 26 035)	1·4% (−3·8 to 6·4)
Cape Verde	67 017 (62 859 to 71 163)	−5·5% (−8·0 to −3·0)	2828 (1789 to 3958)	−3·5% (−7·2 to 0·3)	137 081 (121 467 to 154 885)	−9·0% (−11·9 to −6·1)	445 (280 to 664)	−2·5% (−7·6 to 3·1)
Chad	1 523 050 (1 428 102 to 1 617 545)	−1·6% (−4·2 to 1·3)	62 328 (39 362 to 88 327)	−0·4% (−4·2 to 3·5)	3 373 712 (2 993 397 to 3 801 564)	−1·6% (−4·5 to 1·2)	9748 (6049 to 14 901)	0·9% (−3·9 to 5·7)
Côte d'Ivoire	2 571 760 (2 418 908 to 2 736 143)	0·2% (−2·3 to 2·9)	106 293 (67 451 to 150 428)	2·0% (−1·8 to 5·7)	5 497 208 (4 897 336 to 6 207 625)	−0·8% (−3·5 to 2·2)	16 656 (10 415 to 25 119)	2·7% (−2·0 to 8·1)
The Gambia	221 140 (207 744 to 234 781)	0·8% (−1·7 to 3·8)	9 84 (5 61 to 12 840)	1·7% (−2·0 to 5·6)	481 184 (430 104 to 542 602)	0·6% (−2·1 to 3·6)	1 18 (879 to 2 33)	2·8% (−2·0 to 8·0)
Ghana	3 249 877 (3 046 627 to 3 438 538)	−0·3% (−3·1 to 2·3)	136 143 (87 026 to 192 236)	1·3% (−2·4 to 4·9)	6 764 987 (6 001 684 to 7 607 359)	−1·5% (−4·4 to 1·4)	21 357 (13 352 to 32 032)	2·3% (−2·4 to 7·7)
Guinea	1 450 515 (1 361 218 to 1 539 945)	−1·7% (−4·2 to 1·0)	59 790 (38 449 to 85 019)	−0·2% (−3·7 to 3·4)	3 176 932 (2 842 949 to 3 559 576)	−1·7% (−5·1 to 1·4)	9438 (5924 to 14 199)	1·0% (−3·8 to 6·0)
Guinea-Bissau	216 496 (203 476 to 230 130)	−1·6% (−4·4 to 1·0)	8929 (5669 to 12 625)	−0·4% (−4·0 to 3·5)	472 228 (420 380 to 529 458)	−2·3% (−5·1 to 0·5)	1410 (878 to 2112)	0·7% (−3·7 to 6·1)
Liberia	523 128 (490 307 to 554 766)	−1·6% (−4·2 to 0·9)	21 394 (13 569 to 30 321)	0·1% (−3·8 to 3·9)	1 141 762 (1 021 139 to 1 280 295)	−1·7% (−4·5 to 1·3)	3385 (2124 to 5112)	1·4% (−3·2 to 6·2)
Mali	1 915 462 (1 799 502 to 2 026 148)	−2·5% (−5·1 to 0·3)	78 717 (50 554 to 111 259)	−0·4% (−4·2 to 3·7)	4 270 772 (3 825 614 to 4 776 282)	−3·0% (−5·8 to −0·4)	12 373 (7680 to 18 858)	0·8% (−3·8 to 5·9)
Mauritania	476 034 (446 459 to 504 230)	−2·5% (−5·2 to 0·0)	19 871 (12 731 to 27 981)	−0·8% (−4·5 to 3·1)	996 788 (888 183 to 1 118 703)	−3·9% (−6·9 to −1·0)	3116 (1969 to 4686)	0·2% (−4·4 to 5·4)
Niger	2 091 231 (1 959 929 to 2 214 577)	−2·0% (−4·5 to 0·5)	86 090 (54 389 to 122 758)	−0·7% (−4·3 to 2·9)	4 735 150 (4 249 339 to 5 331 112)	−0·9% (−3·6 to 2·0)	13 571 (8498 to 20 664)	0·9% (−3·6 to 5·8)
Nigeria	20 102 348 (19 040 571 to 21 198 231)	−1·8% (−4·1 to 0·8)	836 053 (535 322 to 1 176 086)	−0·1% (−3·6 to 4·0)	42 325 359 (37 780 915 to 47 530 187)	−3·5% (−6·2 to −0·7)	130 870 (81 828 to 196 851)	0·7% (−4·2 to 6·0)
São Tomé and Príncipe	21 799 (20 434 to 23 145)	−2·0% (−4·8 to 0·6)	909 (579 to 1294)	−0·7% (−4·2 to 3·2)	46 418 (41 228 to 52 354)	−3·0% (−5·8 to 0·0)	142 (90 to 213)	0·5% (−4·0 to 5·9)
Senegal	1 729 552 (1 624 366 to 1 831 698)	−0·3% (−3·1 to 2·3)	71 629 (45 475 to 101 959)	0·9% (−3·1 to 4·7)	3 758 765 (3 366 554 to 4 202 726)	−0·7% (−3·5 to 2·5)	11 215 (7030 to 16 958)	2·0% (−2·4 to 6·9)
Sierra Leone	751 211 (703 266 to 796 776)	−1·2% (−4·0 to 1·3)	31 036 (19 779 to 44 054)	0·1% (−3·7 to 3·7)	1 628 771 (1 441 480 to 1 824 155)	−2·1% (−5·0 to 0·8)	4865 (3057 to 7319)	1·3% (−3·4 to 6·6)
Togo	849 307 (796 127 to 903 033)	−1·6% (−4·4 to 0·9)	35 269 (22 304 to 50 067)	0·2% (−3·6 to 3·9)	1 822 412 (1 622 394 to 2 043 937)	−2·8% (−5·9 to 0·4)	5521 (3430 to 8235)	1·3% (−3·4 to 6·2)
**Eastern sub-Saharan Africa**	**35 406 307 (33 581 801 to 37 179 036)**	**−2·1% (−2·9 to −1·2)**	**1 521 377 (966 800 to 2 138 404)**	**0·0% (−1·5 to 1·3)**	**77 059 874 (68 504 260 to 87 437 615)**	**−11·2% (−12·4 to −9·9)**	**255 465 (161 337 to 377 559)**	**−2·2% (−5·5 to 1·3)**
Burundi	1 058 994 (990 051 to 1 129 267)	−1·8% (−4·6 to 0·9)	45 360 (29 050 to 64 184)	0·1% (−3·4 to 3·9)	2 419 541 (2 147 315 to 2 748 254)	−7·9% (−11·0 to −4·6)	7677 (4781 to 11 540)	−1·4% (−6·5 to 4·4)
Comoros	77 572 (72 661 to 82 472)	−2·3% (−4·8 to 0·3)	3357 (2116 to 4758)	−0·1% (−4·1 to 4·4)	168 749 (148 553 to 190 784)	−11·2% (−13·8 to −8·2)	567 (356 to 842)	−2·1% (−7·5 to 4·2)
Djibouti	94 816 (88 900 to 100 544)	−2·6% (−5·5 to 0·1)	4119 (2626 to 5753)	−0·9% (−4·5 to 2·7)	207 419 (183 060 to 234 817)	−12·5% (−15·2 to −9·4)	706 (447 to 1058)	−3·2% (−8·5 to 3·1)
Eritrea	508 641 (474 930 to 541 485)	−2·8% (−5·4 to −0·3)	21 853 (14 069 to 30 946)	−0·4% (−4·3 to 3·5)	1 130 826 (1 000 222 to 1 285 282)	−12·0% (−15·0 to −9·0)	3689 (2320 to 5510)	−2·4% (−7·8 to 4·0)
Ethiopia	9 229 249 (8 780 775 to 9 658 414)	−1·2% (−3·5 to 1·2)	401 119 (254 815 to 560 661)	1·1% (−2·7 to 5·1)	17 741 436 (15 603 305 to 20 337 447)	−15·1% (−19·3 to −11·2)	64 633 (41 347 to 97 318)	−3·1% (−9·1 to 3·6)
Kenya	4 461 186 (4 252 409 to 4 661 140)	−2·4% (−2·9 to −1·9)	192 821 (123 631 to 270 326)	−1·0% (−1·9 to 0·0)	10 067 590 (8 908 707 to 11 433 434)	−11·0% (−11·9 to −10·1)	33 220 (20 978 to 49 112)	−2·6% (−5·4 to 0·5)
Madagascar	2 374 755 (2 222 187 to 2 536 836)	−1·3% (−3·9 to 1·4)	102 173 (65 469 to 144 565)	0·6% (−3·3 to 4·4)	5 283 896 (4 671 836 to 6 016 456)	−8·6% (−11·7 to −5·7)	17 325 (10 894 to 25 710)	−1·0% (−6·2 to 5·0)
Malawi	1 606 835 (1 502 878 to 1 712 573)	−2·9% (−5·5 to 0·0)	68 422 (43 541 to 97 676)	−1·1% (−4·9 to 2·8)	3 628 261 (3 197 219 to 4 114 340)	−10·1% (−13·0 to −7·3)	11 551 (7305 to 17 329)	−2·5% (−7·7 to 3·6)
Mozambique	2 629 177 (2 461 306 to 2 797 480)	−3·1% (−5·7 to −0·5)	111 324 (70 810 to 157 682)	−1·5% (−5·4 to 2·4)	5 946 894 (5 278 550 to 6 719 065)	−10·5% (−13·1 to −7·7)	18 844 (11 929 to 27 700)	−2·8% (−7·9 to 3·4)
Rwanda	1 172 540 (1 096 165 to 1 251 213)	−0·9% (−3·6 to 1·9)	50 301 (31 976 to 70 958)	1·0% (−2·9 to 5·6)	2 565 086 (2 258 665 to 2 912 265)	−11·8% (−14·6 to −8·5)	8450 (5236 to 12 658)	−1·8% (−7·3 to 4·9)
Somalia	989 744 (924 459 to 1 051 398)	−1·3% (−3·9 to 1·5)	42 078 (27 076 to 59 527)	0·0% (−3·8 to 4·0)	2 277 745 (2 009 536 to 2 584 784)	−7·5% (−10·3 to −4·6)	7156 (4481 to 10 831)	−1·3% (−6·1 to 4·2)
South Sudan	1 220 526 (1 141 159 to 1 300 220)	−1·7% (−4·4 to 0·8)	51 468 (33 039 to 73 017)	1·2% (−2·8 to 5·3)	2 870 947 (2 559 641 to 3 236 742)	−8·5% (−11·0 to −5·6)	8871 (5552 to 13 259)	−0·8% (−5·6 to 5·0)
Tanzania	4 763 051 (4 529 109 to 4 988 380)	−2·5% (−4·7 to −0·3)	206 249 (131 056 to 289 453)	−0·3% (−4·0 to 3·6)	10 878 846 (9 601 348 to 12 327 415)	−8·5% (−12·2 to −4·0)	35 827 (22 485 to 53 261)	−0·9% (−6·1 to 5·3)
Uganda	3 521 034 (3 284 393 to 3 751 194)	−2·7% (−5·3 to −0·2)	149 693 (95 656 to 212 957)	0·2% (−4·0 to 4·2)	7 878 653 (6 987 534 to 9 055 646)	−11·9% (−15·0 to −9·0)	25 070 (15 778 to 37 521)	−1·9% (−7·4 to 4·5)
Zambia	1 674 732 (1 596 596 to 1 752 842)	−1·8% (−3·9 to 0·1)	70 027 (44 394 to 98 653)	−1·0% (−4·4 to 2·6)	3 943 390 (3 517 628 to 4 390 941)	−7·6% (−11·0 to −3·4)	11 708 (7238 to 17 784)	−1·9% (−6·5 to 3·7)
**Central sub-Saharan Africa**	**11 692 589 (11 010 516 to 12 369 766)**	**−1·3% (−3·1 to 0·5)**	**487 602 (310 136 to 687 809)**	**0·6% (−2·0 to 3·2)**	**26 207 351 (23 356 289 to 29 570 790)**	**−3·3% (−5·2 to −1·3)**	**80 144 (50 424 to 120 657)**	**0·6% (−2·5 to 4·5)**
Angola	2 487 245 (2 343 446 to 2 633 502)	−2·1% (−4·7 to 0·2)	104 491 (65 982 to 146 917)	−0·2% (−3·8 to 3·7)	5 461 252 (4 866 498 to 6 175 196)	−7·0% (−9·8 to −3·8)	16 978 (10 636 to 25 688)	−0·6% (−5·8 to 5·2)
Central African Republic	543 715 (510 448 to 575 835)	−1·0% (−3·5 to 1·6)	22 595 (14 527 to 31 973)	0·4% (−3·1 to 4·5)	1 229 346 (1 090 885 to 1 385 696)	−2·9% (−6·0 to 0·2)	3760 (2352 to 5688)	0·5% (−4·0 to 5·5)
Congo (Brazzaville)	478 926 (451 004 to 507 643)	−1·9% (−4·7 to 0·7)	20 205 (13 032 to 28 445)	0·1% (−3·5 to 3·9)	1 018 864 (900 642 to 1 153 466)	−6·1% (−9·1 to −3·1)	3312 (2057 to 4925)	−0·1% (−4·9 to 4·9)
Democratic Republic of the Congo	7 907 386 (7 433 746 to 8 374 225)	−0·9% (−3·4 to 1·6)	328 595 (208 899 to 463 527)	0·9% (−2·6 to 4·8)	17 930 616 (15 956 310 to 20 188 762)	−1·7% (−4·6 to 1·0)	54 169 (34 017 to 81 520)	1·1% (−3·3 to 6·2)
Equatorial Guinea	89 144 (84 153 to 94 177)	−3·7% (−6·1 to −1·2)	3802 (2430 to 5336)	−0·8% (−4·6 to 3·2)	181 012 (160 849 to 205 643)	−14·1% (−17·0 to −11·3)	622 (396 to 921)	−2·5% (−8·3 to 4·2)
Gabon	186 172 (175 149 to 197 012)	−3·2% (−5·7 to −0·6)	7915 (5067 to 11 073)	−1·7% (−5·5 to 2·3)	386 261 (342 337 to 435 059)	−7·5% (−10·5 to −4·5)	1303 (824 to 1926)	−1·5% (−6·3 to 4·0)

95% uncertainty intervals are in parentheses. YLDs=years of life lived with disability. SDI=Socio-demographic Index.

Migraine was estimated to have caused 45·1 million (95% UI 29·0–62·8) YLDs in 2016 (table), an increase of 51·2% (49·7–52·8) from the 29·8 million (19·1–41·8) YLDs in 1990 (see online results tool). Tension-type headache caused 7·2 million (4·6–10·5) YLDs in 2016 (table), an increase of 53·1% (47·5–58·4) from the 4·7 million (3·0–7·0) YLDs in 1990 (online results tool). Together, in 2016, migraine and tension-type headache caused 52·3 million (34·5–72·5) YLDs globally, which was 6·5% of all YLDs: 7·7% among women and 5·1% among men. The global age-standardised YLD rates per 100 000 population for migraine were 598·7 (95% UI 385·9–833·3). The change from the 1990 values was –0·2% (–0·8 to 0·4). For tension-type headache, the global age-standardised YLD rate per 100 000 population was 95·9 (61·5–140·0), a change of –0·2% (–2·5 to 1·9) from 1990 values. The age-standardised YLD rates in 2016 of both disorders were higher in women (migraine 777·6, 500·4–1083·6; tension-type headache 114·6, 73·6–162·4) than in men (migraine 422·3, 274·3–586·7; tension-type headache 77·4, 49·6–113·2). The percentage of all YLDs due to migraine were 5·6% (4·0–7·2) overall: 6·8% (4·9–8·8) for women and 4·3% (3·1–5·5) for men. The corresponding values for tension-type headache were 0·9% (0·7–1·2) overall: 1·0% (0·7–1·3) for women and 0·8% (0·6–1·0) for men.

**Figure 3:**
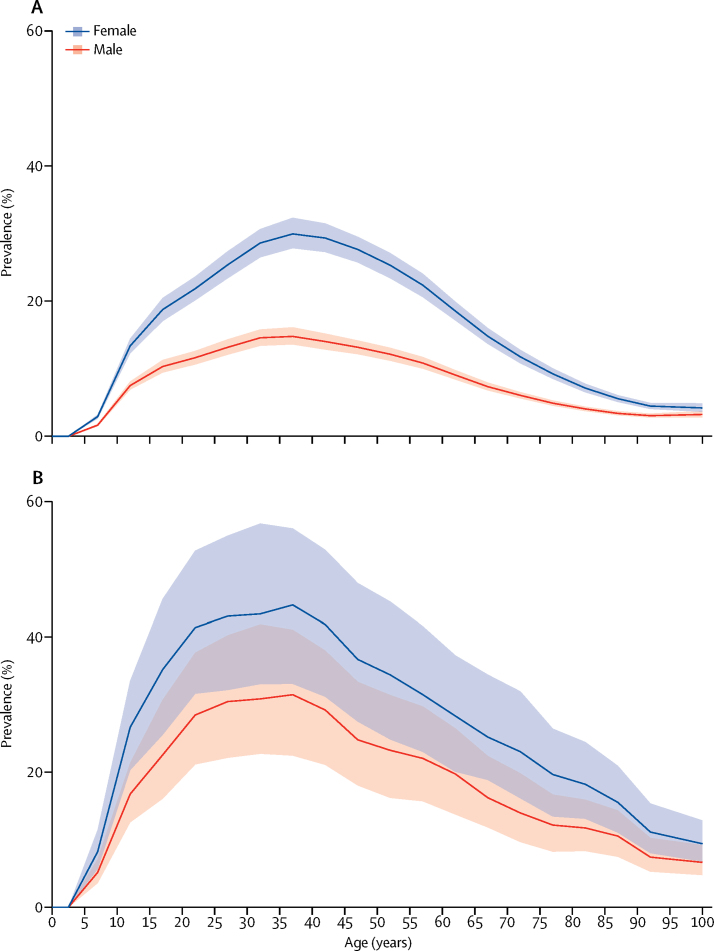
Global prevalence of (A) migraine and (B) tension-type headache by age and sex, 2016 Prevalence is expressed as the percentage of the population that is affected by the disease. Shaded areas show 95% uncertainty intervals. Values are plotted at the midpoint of 5-year age categories.

**Figure 4:**
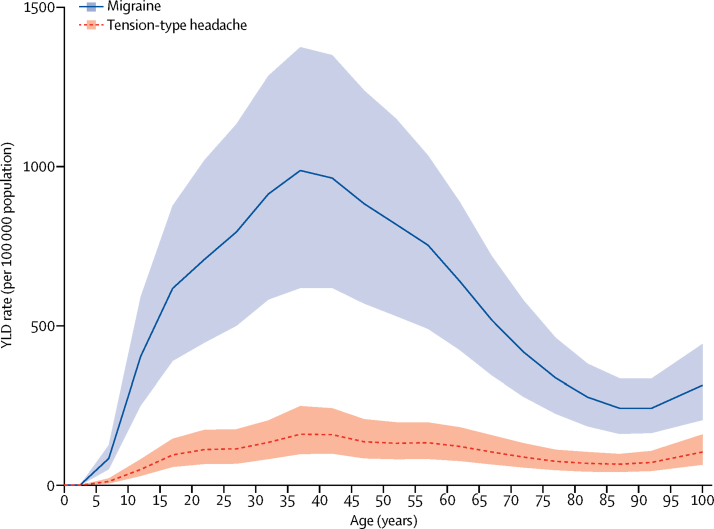
Global years of life lived with disability (YLD) rate per 100 000 population due to migraine and tension-type headache by age, 2016 Shaded areas show 95% uncertainty intervals. Values are plotted at the midpoint of 5-year age categories.

For both migraine and tension-type headache, a peak in prevalence and YLD rate occurred between ages 35 and 39 years (Figure 3, Figure 4). In both sexes, the percentages of all YLDs were highest in the group aged 15–49 years (migraine 8·2%, tension-type headache 1·3%), but was also high in children aged 5–14 years (migraine 4·5%, tension-type headache 0·6%), in individuals aged 50–69 years (migraine 4·2%, tension-type headache 0·7%), and in the elderly (ie, ≥70 years; migraine 1·3%, tension-type headache 0·3%). In women between ages 15 and 49 years, migraine caused 20·3 million (95% UI 12·9–28·5) and tension-type headache caused 2·9 million (95% UI 1·8–4·2) YLDs in 2016, which together were 11·2% of all YLDs in this age and sex group.

The proportions of all DALYs for both sexes were 1·9% (1·3–2·5) for migraine and 0·3% (0·2–0·4) for tension-type headache. The values were 2·7% (1·8–3·6) and 0·4% (0·3–0·5) for women, and 1·2% (0·8–1·7) and 0·2% (0·3–0·3) for men. Globally, migraine ranked 12th of all disorders at GBD cause Level 4 in terms of DALYs for both sexes in 2016, up from 17th in 1990. In 2016, migraine ranked fifth for women (up from 13th in 1990), and 20th for men (up from 28th in 1990).

**Figure 5:**
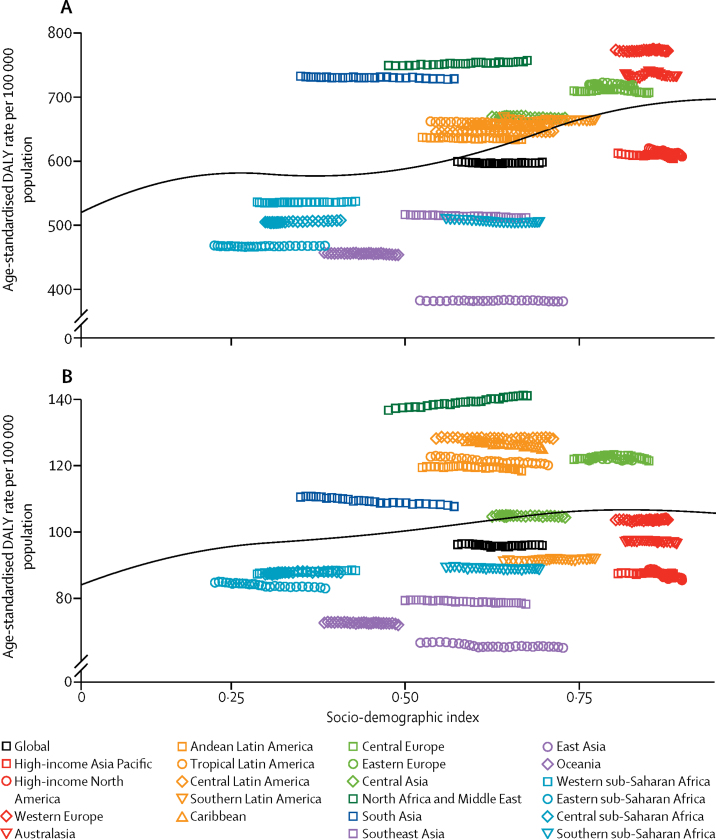
Age-standardised disability-adjusted-life-years (DALY) rates for (A) migraine and (B) tension-type headache by 21 Global Burden of Disease regions by Socio-demographic Index (SDI), 1990–2016 The solid black line represents expected values based on SDI from a regression of all location data over the entire 1990 to 2016 estimation period.

Migraine was among the top five Level 4 causes of age-standardised YLDs in all five SDI quintiles both in 1990 and 2016. Age-standardised DALYs for each headache type showed a small increase as SDI increased. The values estimated for the 21 world regions between 1990 and 2016 varied little over time, but markedly between regions (figure 5). These results indicate that the SDI level of the country is not a major determinant of the size of the headache burden.

## Discussion

Almost three billion people had a migraine and tension-type headache together in 2016; tension-type headache was the third most prevalent disorder (after dental caries and latent tuberculosis infection) and migraine the sixth most prevalent out of 328 diseases and injuries for which GBD 2016 made estimates.^20^

GBD 2016 shows that headache, and in particular, migraine, is one of the main causes of disability worldwide, particularly in young adult and middle-aged women (figure 3). Globally, migraine and tension-type headache together accounted for 6·5% of all YLDs: 7·7% among women and 5·1% among men. Headache is particularly burdensome in people aged 15–49 years, being the cause of 9·5% of all YLDs in this group (11·2% among women and 7·5% among men) but the effect is not negligible in the group aged 5–14 years (5·1%), or among the group aged 50–69 years (4·9%), and the group aged 70 years or older (1·6%). Together the two disorders are the cause of more than 2% of all DALYs: almost 3% in women and almost 1·5% in men.

When analysed at cause Level 4, migraine was the second cause of disability after low back pain and ranked among the top ten causes of YLDs (age-standardised) in all 195 countries, both in 1990 and in 2016.^20^ In 2016, it ranked first in two regions, second in nine, third in five, fourth in two, fifth in two, and sixth in one region. Globally, tension-type headache ranked 28th in both 1990 and 2016.

The increase in YLDs between 1990 and 2016 reflects population growth and potentially some effect of a change in the age composition of many populations (ie, fewer children and adolescents and more middle-aged people). The fact that age-standardised YLD rates are unchanged indicates that, overall, the underlying causes of headache have remained the same. Any improvements in the efficacy of headache treatments during this period (which saw the introduction of triptans for migraine) have had no detectable effect—perhaps partly a reflection of how poorly available they are worldwide^21^ and of the scarcity of data on the proportion of time patients are symptomatic, which did not allow us to find a treatment effect over time and by location. In the DALY rankings, headaches are higher in 2016 than in 1990, because of an overall decrease in YLLs from fatal disorders. In general, DALY rates for non-communicable diseases increase rapidly with age;^22^ hence, counts of deaths and DALYs from non-communicable diseases will increase in ageing populations. However, ageing has a lesser effect on headaches as these, and in particular migraine, are most common among young and middle-aged adults, and become less prevalent with old age.

The principal reason migraine has climbed from the seventh largest cause of YLDs in GBD 2015 to second place in GBD 2016^20^, ^23^ is that medication overuse headache used to be treated as a separate disease entity, but in GBD 2016 it was considered a sequela of either migraine or tension-type headache, with more than 70% of medication overuse headache burden being reattributed to migraine. We believe this change in the status of medication overuse headache is correct, from both clinical and pathophysiological viewpoints, because medication overuse headache virtually never develops de novo, but almost always from either migraine or tension-type headache, and the presence of a primary headache is considered to be a prerequisite for medication overuse headache.^10^, ^24^

A short supply of data is still a limitation in headache burden estimates. Although several epidemiological studies on headache have been done in the past decade in large and populous regions of the world where none existed before (ie, Russia, China, India, and parts of Africa), the majority of studies are still from high-income countries, and there remain five regions of low-income and middle-income countries without data for migraine. No data exist for populous countries like Indonesia, Vietnam, Bangladesh, Egypt, South Africa, and the Democratic Republic of Congo. In sub-Saharan Africa, only five countries have data on headache. For tension-type headache and medication overuse headache, the data are even scarcer. In addition, too few population-based studies give good estimates of average headache duration and frequency, which are necessary for calculation of time spent in the symptomatic state. The small number of studies did not allow us to differentiate the estimates of time spent with symptoms by location, although, in countries with better access to health care, the frequency and duration of migraine attacks would be expected to decrease with treatment. There is also a need to update results in many places, to get more reliable results (many old studies are of low quality) and possibly also to monitor secular trends in headache epidemiology. Because data on headache are poorly captured in national health surveys, and administrative data collections such as those from claims or primary care are unreliable measures of prevalence (appendix), it is desirable that surveys on headache be done in many more places, and preferably at regular intervals as part of national health reporting systems.

In GBD, DisMod-MR 2.1 makes it possible to adjust results of studies that were done using suboptimal case definitions or methods. However, an important aspect that has not yet been taken into account in the model is the distinction between definite and probable diagnoses of migraine and tension-type headache. People with a definite diagnosis fulfil all ICHD diagnostic criteria for the disorder, whereas those with probable diagnoses do not meet one of them; the probable diagnosis is not meant to signify a separate nosological entity. In a clinical setting, probable diagnoses are useful for patients for whom there is diagnostic uncertainty, but confirmation (or refutation) of the diagnosis is expected later. However, this will not be the case in epidemiological studies and, unless the large number of probable cases are included for either migraine or tension-type headache as appropriate, these people will not be counted, despite experiencing headache-related disability. Several studies have investigated, in more detail, participants in epidemiological studies who received probable diagnoses. In the great majority, a definite diagnosis of migraine was withheld because attacks reportedly lasted less than 4 h (the minimum allowed by the criteria).^25^, ^26^, ^27^ Otherwise, the diagnosis resembled migraine closely, and was associated with considerable disability. Until 2000, the majority of studies reported only definite migraine, and not both definite and probable diagnoses. After 2000, more studies reported both types, and these studies indicate that the prevalence of probable migraine is almost as high as that of definite migraine.^26^, ^28^ Future iterations of GBD should therefore find a reasonable and consistent way to deal with probable migraine because it is a common cause of disability that otherwise will be unaccounted for. Similar arguments can be made for probable tension-type headache, although knowledge of this headache type is far less, and the YLDs missed by omitting this are fewer because of the much lower disability weight of tension-type headache compared with migraine.

Another problem is the handling of chronic headache disorders (ie, present on 15 or more days per month for more than 3 months). Many of these headaches will fall into the categories of chronic migraine, chronic tension-type headache, or medication overuse headache, but the high frequency of headaches tends to blur the features necessary for diagnosis. These headaches become even more difficult to diagnose with certainty in cross-sectional epidemiological studies based on questionnaires, especially when these are self-administered or applied by lay interviewers. With the exception of medication overuse headache, which is diagnosed by enquiry into medication use, these headaches are usually lumped into a descriptive category of headache on 15 or more days per month, and not counted in GBD. Hence, there might be additional, and perhaps considerable, headache-related morbidity that is not captured by the diagnoses used in GBD.

Despite efforts to adjust for methodological differences between studies, part of the variation between countries might be due to residual measurement error, rather than true variation. Efforts have been made to standardise the methods for studies on prevalence and burden of headache,^11^ and in the future adherence to these will hopefully make it easier to compare the burden over geographical borders and time periods. One of the greater challenges in modelling headache is the poor knowledge of predictors. Such predictors help to stabilise disease models in the sense that they adjust data points that are affected by measurement error.

Another problem concerns how to estimate the proportion of time during which people with a headache disorder actually have headache (time in symptomatic state). This difficulty exists partly because average duration and frequency of headaches are usually reported in categories, and estimates thus depend on choice of mean value in each category, and partly because the present figures rely on older studies that are of rather poor general quality. Better estimates can probably be obtained with a more systematic analysis of individual record data in some of the major surveys done in the past 10 years that are of higher quality than earlier studies.

Despite these methodological challenges, headache disorders are ubiquitous and contribute to a large burden of lost health. Sizeable resources would be needed to prevent or alleviate this burden in the hundreds of millions of people with headache worldwide. Until now, most interventions have aimed at the management of symptoms, but preferable for such an immense public health problem might be modification at a population level of risk factors, if such can be identified. This intervention would require greater knowledge of the modifiable factors that drive headache: several have been suggested with varying degrees of scientific support, such as obesity, smoking, indoor and outdoor air pollution, level of physical activity, altitude, blood pressure, and level of stress.^29^, ^30^, ^31^, ^32^ Of these factors, altitude and stress are not included as risks in GBD. For the other postulated risks, the evidence for an effect on headaches is, in our opinion, insufficient. Potential headache risk factors should be tested against GBD causal criteria for inclusion of risks and associated outcomes.^33^ If none of these risk factors pass the criteria, more and better epidemiological and pathophysiological studies should be done to prove or refute hypotheses about causation.

Headache disorders do not appear to be strongly linked to socioeconomic development, as measured by SDI (table). The previous notion that headache was mainly a disorder of high-income countries and particularly prevalent among the wealthy, is refuted by the present study, but neither is the opposite true. Hence, no significant reduction in the global burden of headache can be expected from the demographic and epidemiological transitions^22^ that large parts of the world are presently undergoing, because no clear pattern of decreasing YLD rate with increasing SDI exists. It can, however, be predicted that the relative importance of headaches will further increase as the importance of other disorders, such as malnutrition, infections, maternal and child diseases, and cardiovascular and other fatal non-communicable disorders decreases. Although a socioeconomic index like the SDI does not reflect differences in headache prevalence when applied across countries and cultures, the possibility that such factors are important within a country or region cannot be eliminated. A socioeconomic gradient, to the effect that low socioeconomic status is linked to higher headache prevalence, has been shown in countries of high, middle, and low income.^15^, ^34^, ^35^, ^36^

Even if it proves difficult to establish, with reasonable certainty, that modifiable risk factors exist for headache, the results of GBD definitely give a strong call for improving health care for headache. This call involves the inclusion of headache care in existing health-care systems, and not only in the high-income part of the world. Implementation of a headache service in Georgia, a country where none existed previously, has been economically sustainable.^37^ An educational programme among general practitioners in Estonia has been shown to reduce unnecessary referrals to specialists and special examinations.^38^ A programme to increase competence in headache care in China is now being implemented.^39^ Some simple remedies and methods, like aspirin for attack treatment and amitriptyline for prevention of migraine, together with provision of information for patients and education for health-care providers, should be highly cost-effective in low-income and middle-income countries.^40^ A study from European and Latin American countries has shown that discontinuation of medication overuse can reduce the proportion of severely disabled patients with medication overuse headache by almost 60%.^41^ This study also indicates that increased awareness of the danger of non-critical use of acute medication might prevent millions of people from developing this prevalent and disabling disorder.

In high-income parts of the world, the results presented here also highlight a strong moral obligation to allocate more resources to research aimed at understanding the mechanisms of headache to enable development of more effective prevention and treatments. At the same time, current treatments need to be recognised as ineffective more because of poor availability than inefficacy, and health services must do a better job of reaching people if new treatments are to have an effect.^42^ For the pharmaceutical industry, the market for proven cost-effective remedies is huge, as is the potential for a large decrease in pain and disability, and an increase in productivity, for the global community.

In conclusion, major limitations still exist in the GBD headache burden estimations, the most notable being the short supply of epidemiological data from large parts of the world, the paucity of studies giving data on average time with headache, and great methodological heterogeneity. Nevertheless, GBD 2016 confirms that headache, and in particular, migraine, is a large public health problem in both sexes and all age groups worldwide, but most so in young and middle-aged women. Headache is not limited to the high-income part of the world and, unless action is taken, it is here to stay: there is no indication that the demographic and epidemiological transitions alone will improve the situation. Rather, these profound changes which reduce mortality will increase the relative importance of headache for public health.

For **data sources** see http://ghdx.healthdata.org/gbd-2016/data-input-sources
