# Correction to *Lancet Neurol* 2018; 17: 954–76

**DOI:** 10.1016/S1474-4422(21)00380-X

**Published:** 2021-12

*GBD 2016 Headache Collaborators. Global, regional, and national burden of migraine and tension-type headache, 1990–2016: a systematic analysis for the Global Burden of Disease Study 2016.* Lancet Neurol *2018; **17:** 954–76*—In this Article, Ali H Mokdad should have been included in the GBD 2016 Headache Collaborators. The affiliations have also been updated. These corrections have been made to the online version as of Nov 17, 2021.

