# Supplementary material for: Global, regional, and national burden of migraine and tension-type headache, 1990–2016: a systematic analysis for the Global Burden of Disease Study 2016
Source: Lancet Neurol. 2018 Nov;17(11):954–76. doi: 10.1016/S1474-4422(18)30322-3 (PMC6191530; doi:10.1016/S1474-4422(18)30322-3)
Supplement: Supplementary appendix [file mmc1.pdf]

THE LANCET
Neurology

Supplementary appendix

This appendix formed part of the original submission and has been peer reviewed. We post it as supplied by the authors.

Supplement to: GBD 2016 Headache Collaborators. Global, regional, and national burden of migraine and tension-type headache, 1990–2016: a systematic analysis for the Global Burden of Disease Study 2016. *Lancet Neurol* 2018; **17:** 954–76.

Appendix 1

Summary of General Global Burden of Disease Study Methods:

The Institute of Health Metrics and Evaluation with a growing collaboration of scientists produces annual updates of the Global Burden of Disease study. Estimates span the period from 1990 to the most recent completed year. By the time of the release of GBD 2016 in September 2017, there were over 2,700 collaborators in 132 countries who contributed to this global public good. Annual updates allow incorporation of new data and method improvements to ensure that the most up-to-date information is available to policy makers in a timely fashion to help make resource allocation decisions. In this analysis, we have aggregated results from GBD 2016 for 15 disease and injury outcomes that are generally cared for by neurological services. These include infectious conditions (tetanus, meningitis, encephalitis), stroke, brain and other nervous system cancers, traumatic brain injury and spinal cord lesion which are classified outside the more narrowly defined category of neurological disorders in GBD (i.e., Alzheimer disease and other dementias, Parkinson disease, multiple sclerosis, motor neuron disease, idiopathic epilepsy, migraine, tension-type headache and a rest category of less common other neurological disorders). Compared to a previous analysis based on GBD 2015,[1] we were able to add the non-fatal outcomes of traumatic brain injury and spinal cord lesion and medication overuse headache is no longer included as a separate cause but quantified as a consequence of the underlying headache types.

In the methods section of this overview paper we present a summary of the general methods of the global burden of disease. In the accompanying disease-specific papers we concentrate on methods that are specific to each disorder. The guiding principle of GBD is to assess health loss due to mortality and disability comprehensively where we define disability as any departure from full health. In GBD2016, estimates were made for 195 countries and territories, and 579 subnational locations, for 27 years starting from 1990, for 23 age groups and both sexes. Deaths were estimated for 264 disease and injuries while prevalence and incidence were estimated for 328 diseases and injuries. In order to allow meaningful comparisons between deaths and non-fatal disease outcomes as well as between diseases, the data on deaths and prevalence are summarized in a single indicator, the disability-adjusted life year (DALY). DALYs are the sum of years of life lost (YLLs) and years lived with disability (YLDs). YLLs are estimated as the multiplication of counts of death and a standard, 'ideal', remaining life expectancy at the age of death. The standard life expectancy is derived from the lowest observed mortality rates in any population in the world greater than 5 million.[2] YLDs are estimated as the product of prevalence of individual consequences of disease (or 'sequelae') times a disability weight that quantifies the relative severity of a sequela as a number between zero (representing 'full health') and 1 (representing death). Disability weights have been estimated in nine population surveys and an open-access internet survey in which respondents are asked to choose the 'healthier'[3] between random pairs of health states that are presented with a short description of the main features.

All-cause mortality rates are estimated from vital registration data in countries with complete coverage. For other countries, the probabilities of death before age 5 and between ages 15 and 60 are estimated from censuses and surveys asking mothers to provide a history of children ever born and those still alive, and surveys asking adults about siblings who are alive or have passed away. Using model life tables, these probabilities of death are transformed into age-specific death rates by location, year and sex. GBD has collated a large database of cause of death data from vital registrations and verbal autopsy surveys in which relatives are asked a standard set of questions to ascertain the likely cause of death, supplemented with police and mortuary data for injury deaths in

countries with no other data. For countries with vital registration data, the completeness is assessed with demographic methods based on comparing recorded deaths with population counts between two successive censuses. The cause of death information is provided in a large number of different classification systems based on versions of the International Classification of Diseases or bespoke classifications in some countries. All data are mapped into the disease and injury categories of GBD. All classification systems contain codes that are less informative because they lack a specific diagnosis (e.g. unspecified cancer) or refer to codes that cannot be underlying cause of death (e.g., low back pain or senility) or are intermediate causes (e.g., heart failure or sepsis). Such deaths are redistributed to more precise underlying causes of death.[4] After these redistributions and corrections for under-registration the data are analysed in CODEm (cause of death ensemble model), a highly systematized tool that runs many different models on the same data and chooses an ensemble of models that best reflects all the available input data. Models are chosen with variations in the statistical approach ('mixed effects' of space-time Gaussian Process Regression), in the unit of analysis (rates or cause fractions), and the choice of predictive covariates. The statistical performance of all models is tested by holding out 30% of the data and checking how well a model covers the data that were held out. To enforce consistency from CODEm, the sum of all cause-specific mortality rates is scaled to that of the all-cause mortality rates in each age, sex, location and year category.

Non-fatal estimates are based on systematic reviews of published papers and unpublished documents, survey microdata, administrative records of health encounters, registries and disease surveillance systems. Our Global Health Data Exchange (GHDx, http://ghdx.healthdata.org/) is the largest repository of health data globally. We first set a reference case definition and/or study method that best quantifies each disease or injury or consequence thereof. If there is evidence of a systematic bias in data that used different case definitions or methods compared to reference data we adjust those data points to reflect what its value would have been if measured as the reference. This is a necessary step if one wants to use all data pertaining to a particular quantity of interest rather than choosing a small subset of data of the highest quality only. DisMod-MR 2.1, a Bayesian meta-regression tool, is our main method of analyzing non-fatal data. It is designed as a geographical cascade where a first model is run on all the world's data which produces an initial global fit and estimates coefficients for predictor variables and the adjustments for alternative study characteristics. The global fit adjusted by the values of random effects for each of 7 GBD super-regions, the coefficients on sex and country predictors, are passed down as data to a model for each super-region together with the input data for that geography. The same steps are repeated going from super-region to 21 region fits and then to 195 fits by country and where applicable a further level down to subnational units. Below the global fit, all models are run separately by sex and for 6 time periods: 1990, 1995, 2000, 2005, 2010 and 2016. During each fit all data on prevalence, incidence, remission (i.e., cure rate) and mortality are forced to be internally consistent. For most diseases, the bulk of data on prevalence or incidence is at the disease level with fewer studies providing data on the proportions of cases of disease in each of the sequelae defined for the disease. The proportions in each sequela are pooled using DisMod-MR 2.1 or meta-analysis, or derived from analyses of patient-level data sets. The multiplication of prevalent cases for each disease sequela and the appropriate disability weight produces YLD estimates that do not yet take into account comorbidity. To correct for comorbidity, these data are used in a simulation to create hypothetical individuals in each age, sex, location and year combination who experience no, one or multiple sequelae simultaneously. We assume that disability weights are multiplicative rather than additive as this avoids assigning a combined disability weight value in any individual to exceed 1, i.e., be

worse than a 'year lost due to death'. This comorbidity adjustment leads to an average scaling down of disease-specific YLDs ranging from around 2% in young children up to 17% in oldest ages.

All our estimates of causes of death are categorical: each death is assigned to a single underlying cause. This has the attractive property that all estimates add to 100%. For risks, we use a different, 'counterfactual', approach, i.e. answering the question: "what would the burden have been if the population had been exposed to a theoretical minimum level of exposure to a risk". Thus, we need to define what level of exposure to a risk factor leads to the lowest amount of disease. We then analyse data on the prevalence of exposure to a risk and derive relative risks for any risk-outcome pair for which we find sufficient evidence of a causal relationship. Prevalence of exposure is estimated in DisMod-MR 2.1, using space-time Gaussian Process Regression, or from satellite imagery in the case of ambient air pollution. Relative risk data are pooled using meta-analysis of cohort, case-control and or intervention studies. For each risk and outcome pair, we evaluate the evidence and judge if the evidence falls into the categories of 'convincing' or 'probable' as defined by the World Cancer Research Fund.[5] From the prevalence and relative risk results, population attributable fractions are estimated relative to the theoretical minimum risk exposure level (TMREL). When we aggregate estimates for clusters of risks, e.g. metabolic or behavioral risks, we use a multiplicative function rather than simple addition and take into account how much of each risk is mediated through another risk. For instance, some of the risk of high body mass index is directly onto stroke as an outcome but much of its impact if mediated through high blood pressure, high cholesterol or high fasting plasma glucose and we would not want to double count the mediated effects when we estimate aggregates across risk factors.[6]

Uncertainty is propagated throughout all these calculations by creating 1,000 values for each prevalence, death, YLL, YLD or DALY estimate and performing aggregations across causes and locations at the level of each of the 1,000 values for all intermediate steps in the calculation. The lower and upper bounds of the 95% uncertainty interval are the 25th and 975th values of the ordered 1,000 values. For all age-standardised rates, GBD uses a standard population calculated as the non-weighted average across all countries of the percentage of the population in each 5-year age group for the years 2010 to 2035 from the United Nations Population Division's World Population Prospects (2012 revision).[7,8]

GBD uses a composite indicator or sociodemographic development, SDI, which reflects the geometric mean of normalized values of a location's income per capita, the average years of schooling in the population 15 and over, and the total fertility rate. Countries and territories are grouped into 5 quintiles of high, high-middle, middle, low-middle, and low SDI based on their 2016 values.[2]

1 GBD 2015 Neurological Disorders Collaborator Group. Global, regional, and national burden of neurological disorders during 1990-2015: a systematic analysis for the Global Burden of Disease Study 2015. *Lancet Neurol* 2017; **16**: 877–97.

2 GBD 2016 Mortality Collaborators. Global, regional, and national under-5 mortality, adult mortality, age-specific mortality, and life expectancy, 1970-2016: a systematic analysis for the Global Burden of Disease Study 2016. *Lancet Lond Engl* 2017; **390**: 1084–150.

3 Salomon JA, Haagsma JA, Davis A, *et al.* Disability weights for the Global Burden of Disease 2013 study. *Lancet Glob Health* 2015; **3**: e712-723.

4 GBD 2016 Causes of Death Collaborators. Global, regional, and national age-sex specific mortality for 264 causes of death, 1980-2016: a systematic analysis for the Global Burden of Disease Study 2016. *Lancet Lond Engl* 2017; **390**: 1151–210.

5 American Institute for Cancer Research. Food, nutrition, physical activity, and the prevention of cancer: a global perspective. Washington, DC: American Institute for Cancer Research, 2007.

6 GBD 2016 Risk Factors Collaborators. Global, regional, and national comparative risk assessment of 84 behavioural, environmental and occupational, and metabolic risks or clusters of risks, 1990-2016: a systematic analysis for the Global Burden of Disease Study 2016. *Lancet Lond Engl* 2017; **390**: 1345–422.

7 GBD 2013 Mortality and Causes of Death Collaborators. Global, regional, and national age-sex specific all-cause and cause-specific mortality for 240 causes of death, 1990-2013: a systematic analysis for the Global Burden of Disease Study 2013. *Lancet Lond Engl* 2015; **385**: 117–71.

8 United Nations Department of Economics and Social Affairs Population Division. World Population Prospects: The 2012 Revision. http://esa.un.org/unpd/wpp/Documentation/publications.htm (accessed Nov 4, 2014).

Migraine

Case definition

Migraine is a disabling primary headache disorder, typically characterised by recurrent moderate or severe unilateral pulsatile headaches. The two major types are migraine without aura and migraine with aura (transient neurological symptoms). In GBD, we do not distinguish types as most epidemiological studies report on overall migraine only. The ICD-10 code for migraine is G43 and ICD-9 code is 346.

Migraine can give rise to medication overuse headache (MOH), with the following International Classification of Headache Disorders (ICHD-3) diagnostic criteria:

A. Headache occurring on ≥15 days/month in a patient with a pre-existing headache disorder

B. Regular overuse for >3 months of one or more drugs that can be taken for acute and/or symptomatic treatment of headache

C. Not better accounted for by another ICHD-3 diagnosis.

ICHD-3 explicitly states that, when a person fulfils criteria for both migraine and MOH, both diagnoses should be given. However, our GBD headache collaborators, Steiner and Stovner, say that, in survey practice, a screening question on chronic headache is used first, followed by questions to determine if medication overuse is present. This means the diagnoses of migraine and MOH become mutually exclusive (obviating any potential problem of double-counting).

Input data

Search terms on PubMed

Migraine:

(("migraine disorders"[MeSH Terms] OR ("migraine"[All Fields] AND "disorders"[All Fields]) OR "migraine disorders"[All Fields] OR "migraine"[All Fields]) AND ("epidemiology"[Subheading] OR "epidemiology"[All Fields] OR "epidemiology"[MeSH Terms])) AND ("2011/01/01"[PDAT] : "2015/12/31"[PDAT])

Model inputs

Systematic reviews of migraine and MOH were conducted for GBD 2010 and updated for GBD 2013. In GBD 2015, three new representative surveys conducted by GBD collaborators were added in Norway; Karnataka state in India; and Nepal. In GBD 2016, four new representative surveys conducted by GBD collaborators were included for migraine (in Ethiopia, Germany, Denmark, and Sweden), and two for MOH (in Norway and Ethiopia).

Inclusion criteria of the systematic reviews were:

- Representative, population-based surveys
- Reporting of prevalence of migraine headache

In addition, US claims data for 2000, 2010, and 2012 by US state were used.

The table below illustrates the geographic distribution of migraine data.

	Prevalence	Incidence	Remission	Frequency and duration of episodes
Studies	120	4	1	13
GBD world regions	15	2	1	9

The table below shows the geographic distribution of MOH data.

	Prevalence	Incidence	Remission
Studies	28	0	7
GBD world regions	10	0	1

Proportion of Time Symptomatic

To determine the proportion of time over a year spent with migraine headache ("time symptomatic"), 13 studies providing data on the frequency and average duration of episodes were meta-analysed. Since many of these studies reported either or both variables by categories, an assumption was made that the mean represented each category. The pooled estimate from these studies indicated that the time symptomatic was 0.085 (0.058–0.112).

GBD disability weight (DW) survey assessments were based on lay descriptions of sequelae highlighting the major functional consequences and symptoms. The lay description and DW for migraine are shown below.

	Lay description	DW (95% CI)
Migraine	This person has severe, throbbing head pain, and nausea that cause great difficulty in daily activities and sometimes confine the person to bed. Moving around, light, and noise make it worse.	0.434 (0.285–0.603)

Modelling strategy

We used a list of binary covariates which are a modified version of quality indicators of epidemiological studies on headache (Steiner TJ, Stovner LJ et al [2013]. Improving quality in population surveys of headache prevalence, burden, and cost: key methodological considerations. J Headache Pain, 14: 87) and shown in the table below.

Study covariate	Notation	
	Less desirable (1)	Reference (zero)
Other than one-year recall period	Point prevalence	One-year prevalence
Not representative	Selected population	General population or community-based sample from whole country OR general population or community-based sample from defined region within a country, or school-based (for children)
Low-quality sampling method	Not stated OR no (or failed) attempt to secure representativeness	Total defined population, or random sample corrected for population demographics OR random sample uncorrected for population demographics

Poor response	Not stated, or <70%	70–100%
Low-quality survey method and type of interviewer	Not stated OR self-administered (unsupervised) questionnaire OR telephone or face-to-face interview by untrained or unspecified interviewer(s)	Face-to-face interview with headache expert or trained interviewer
Low-quality validation of diagnostic instrument	Instrument not specified or not validated OR validated, but sensitivity and/or specificity <70% OR validated only in screen-positive sub-sample, or in clinic or unspecified sample, but sensitivity and specificity ≥70%	Validated in target population or similar, and sensitivity and specificity ≥70%, or all diagnoses made in face-to-face or telephone interviews by headache expert
Low-quality diagnostic criteria	Not stated OR stated, other than ICHD OR ICHD (or reasonable modification)	ICHD (or reasonable modification)

We added separate covariates for lifetime recall and three years of claims data from MarketScan (2000, 2010, and 2012).

Prior settings in the DisMod model included setting incidence to 0 before age 5 based on expert advice. We also assumed no excess mortality due to migraine. Remission rate was set to be between 0 and 0.1.

All study covariates were initially evaluated as x-cov (which means that data points were adjusted to the reference value if a systematic bias was detected); those that did not have a significant coefficient, were entered as z-cov (which means that a multiplier was applied to the standard error of such data points to indicate they were less certain values because they did not meet the reference criteria for study quality). The table below shows the fixed effect values of the x-covs which are in log space (as DisMod uses an offset lognormal model) as well as the exponentiated values which for an x-cov can be interpreted as an odds ratio.

Only the covariate for low quality survey method and type of interviewer, poor response and three years of claim data remained as x-covs.

The covariates for other than one-year recall period, low-quality sampling method, low-quality diagnostic criteria, low-quality validation of diagnostic instrument, and not representative studies had non-significant coefficients as an x-cov and were subsequently used as z-covs.

Study covariate	Parameter	beta	Exponentiated beta
Low quality survey method and type of interviewer	Prevalence	0.09	1.09 (0.99 – 1.19)
Poor response	Prevalence	- 0.25	0.78 (0.71 – 0.85)
Claims data US 2000	Prevalence	- 2.45	0.086 (0.082 – 0.095)
Claims data US 2010	Prevalence	- 2.07	0.13 (0.12 – 0.14)
Claims data US 2012	Prevalence	- 1.97	0.14 (0.13 – 0.15)

MOH was initially modelled separately in DisMod, then included as a sequela of migraine in the proportion estimated as due to migraine: (73.4%; 95% confidence interval 63.9–82.0%). Therefore, we multiplied MOH prevalence by this factor.

Prior settings in the DisMod model included setting incidence to 0 before age 5 according to expert advice. We also assumed no excess mortality due to MOH. Based on the seven literature sources on remission (listed in references below), we set the bounds of remission to be between 0 and 0.4

All study covariates for MOH were evaluated using the same strategy as modelling for migraine.

The study with recall period other than one year was the only covariate used as a x-covariate; however, its coefficient was insignificant: beta = -0.19 and exponentiated beta = 0.83 (0.66–1.04). The others were subsequently used as z-covariates.

Study covariate	Parameter	beta	Exponentiated beta
Low-quality diagnostic criteria	Prevalence	0.24	1.27 (1.05–1.59)
Poor response	Prevalence	-0.64	0.53 (0.42–0.65)

In GBD 2015, to the prevalence output from DisMod, we first applied the finding from da Silva (2010) that 60% (40.8–79.2%) of "probable" MOH cases were confirmed cases of MOH. However, headache collaborators argued that this would leave the 40% unaccounted for, since surveys first ask about chronic headache and medication overuse before applying criteria for migraine and TTH. Thus, in GBD 2016, we no longer multiplied the prevalence from DisMod by this factor but considered all "probable" cases as MOH cases. We estimated the proportion of time symptomatic, ie, with headache, from the Ayzenberg (2012) estimate of 23.1 days a month with headache and multiplied estimates by 75.9% (72.9–78.8%).

Tension-type Headache

Case definition

Tension-type headache (TTH) is characterised by a dull, non-pulsatile, diffuse, band-like (or vice-like) pain of mild to moderate intensity in the head or neck. The ICD-10 code for TTH is G44.2 and the ICD-9 code is 339.1.

TTH can give rise to medication overuse headache (MOH), with the following International Classification of Headache Disorders (ICHD-3) diagnostic criteria:

A. Headache occurring on ≥15 days/month in a patient with a pre-existing headache disorder
B. Regular overuse for >3 months of one or more drugs that can be taken for acute and/or symptomatic treatment of headache
C. Not better accounted for by another ICHD-3 diagnosis.

ICHD-3 explicitly states that, when a person fulfils criteria for both TTH and MOH, both diagnoses should be given. However, our GBD headache collaborators, Steiner and Stovner, say that, in survey practice, a screening question on chronic headache is used first, followed by questions to determine if medication overuse is present. This means the diagnoses of TTH and MOH become mutually exclusive (obviating any potential problem of double-counting).

Input data

Search terms on PubMed

TTH:

("headache"[MeSH Terms] OR "headache"[All Fields]) AND (tension[All Fields] OR ("pharmaceutical preparations"[MeSH Terms] OR ("pharmaceutical"[All Fields] AND "preparations"[All Fields]) OR "pharmaceutical preparations"[All Fields] OR "medication"[All Fields])) AND ("epidemiology"[Subheading] OR "epidemiology"[All Fields] OR "epidemiology"[MeSH Terms]) AND ("2011/01/01"[PDAT] : "2015/12/31"[PDAT])

Model Inputs

A systematic review of TTH was conducted for GBD 2010 and updated for GBD 2013. In GBD 2015 three new representative surveys conducted by GBD collaborators were added (in Norway, Karnataka State in India, and Nepal. In GBD 2016, two new representative surveys conducted by GBD collaborators were added (in Ethiopia and Germany). Inclusion criteria of the systematic reviews were:

- Representative, population-based surveys
- Reporting of prevalence of TTH headache

In addition, US claims data for 2000, 2010, and 2012 by US state were included.

The table below illustrates the geographic distribution of TTH data.

	Prevalence	Incidence	Remission	Frequency and duration of episodes
Studies	70	1	0	7
GBD world regions	16	1	0	6

The table below shows the geographic distribution of MOH data.

	Prevalence	Incidence	Remission
Studies	28	0	7
GBD world regions	10	0	1

Proportion of Time Symptomatic

To determine the proportion of time over a year spent with the headache of TTH (“time symptomatic”), seven studies providing data on the frequency of episodes and the average duration of episodes were meta-analyzed. Since many of these studies reported either or both variables by categories, an assumption was made that the mean represented each category. The pooled estimate from these studies indicated that the time symptomatic was 0.047 (0.013–0.080).

GBD disability weight (DW) survey assessments were based on lay descriptions of sequelae highlighting the major functional consequences and symptoms. The lay description and DW for TTH are shown below.

	Lay description	DW (95% CI)

TTH	This person has a moderate headache that also affects the neck, which causes difficulty in daily activities	0.036 (0.023–0.053)

Modelling strategy

We used a list of binary covariates which are a modified version of quality indicators of epidemiological studies on headache (Steiner TJ, Stovner LJ et al (2013). Improving quality in population surveys of headache prevalence, burden and cost: key methodological considerations. *J Headache Pain*, 14: 87) and shown in the table below.

Study covariate	Notation	
	Less desirable (1)	Reference (zero)
Other than one-year recall period	Point prevalence	One-year prevalence
Not representative	selected population	general population or community-based sample from whole country OR general population or community-based sample from defined region within a country, or school-based (for children)
Low-quality sampling method	not stated OR no (or failed) attempt to secure representativeness	total defined population, or random sample corrected for population demographics OR random sample uncorrected for population demographics
Poor response	not stated, or <70%	70–100%
Low-quality survey method and type of interviewer	not stated OR self-administered (unsupervised) questionnaire OR telephone or face-to-face interview by untrained or unspecified interviewer(s)	face-to-face interview with headache expert or trained interviewer
Low-quality validation of diagnostic instrument	instrument not specified or not validated OR validated, but sensitivity and/or specificity <70% OR validated only in screen-positive sub-sample, or in clinic or unspecified sample, but sensitivity and specificity ≥70%	validated in target population or similar, and sensitivity and specificity ≥70%, or all diagnoses made in face-to-face or telephone interviews by headache expert
Low-quality diagnostic criteria	not stated OR stated, other than ICHD OR ICHD (or reasonable modification)	ICHD (or reasonable modification)

We added separate covariates for chronic headache, lifetime recall and the three years of claims data from MarketScan (2000, 2010, and 2012).

Prior settings in the DisMod model included setting incidence to 0 before age 5 according to expert advice. We also assumed no excess mortality due to TTH. In the absence of any data on remission, we set bounds between 0 and 0.5, ie, ensuring an average duration of at least two years.

All study covariates were initially evaluated as x-cov (which means that data points were adjusted to the reference value if a systematic bias is detected); those that did not have a significant coefficient

were entered as z-cov (which means that a multiplier was applied to the standard error of such data points to indicate they were less certain values because they did not meet the reference criteria for study quality). The table below shows the fixed-effect values of the x-covs which are in log space (since DisMod uses an offset lognormal model) as well as the exponentiated values which for an x-cov can be interpreted as an odds ratio.

The covariate for other than one-year recall period and not representative had non-significant coefficients as an x-cov and were subsequently used as a z-cov.

Study covariate	Parameter	beta	Exponentiated beta
Chronic headache	Prevalence	-0.37	0.69 (0.41 – 0.98)
Recall lifetime	Prevalence	0.74	2.09 (1.36 – 2.69)
Claims data US 2000	Prevalence	- 4.49	0.011 (0.011 – 0.011)
Claims data US 2010	Prevalence	- 4.12	0.016 (0.016 – 0.017)
Claims data US 2012	Prevalence	- 4.00	0.018 (0.018 – 0.019)

The very low coefficients in claims data meant that few cases of TTH were included in claims data. Data points were crosswalked up by a factor of 50 or more. We decided to include the data with such large crosswalks as we had no other data for the states of the USA and the crosswalks estimated by DisMod were within range of the data from three US studies in Massachusetts, Maryland, and Kentucky.

MOH was initially modelled separately in DisMod, then included as a sequela of TTH in the proportion estimated as due to TTH: 26.6% (18.0–36.1%). Prior settings in the DisMod model included setting incidence to 0 before age 5 according to expert advice. We also assume no excess mortality due to MOH. Based on seven literature sources on remission (listed in references below), we set the bounds of remission to be between 0 and 0.4.

All study covariates for MOH were evaluated using the same strategy as modelling for TTH.

The study with recall period other than one year was the only covariate used as an x-covariate; however, its coefficient was insignificant: beta = -0.19 and exponentiated beta = 0.83 (0.66–1.04). The others were subsequently used as z-covariates.

In GBD 2015, to the prevalence output from DisMod, we first applied the finding from da Silva (2010) that only 60% (40.8–79.2) of “probable” MOH cases were confirmed cases of MOH. However, headache collaborators argued that this would leave the 40% unaccounted for, since surveys first ask about chronic headache and medication overuse before applying criteria for migraine and TTH. Thus, in GBD 2016, we no longer multiplied the prevalence from DisMod by this factor but considered all “probable” cases as MOH cases. We estimated the proportion of time symptomatic, i.e., with headache, from the Ayzenberg (2012) estimate of 23.1 days a month with headache and multiplied estimates by 75.9% (72.9–78.8).

GATHER checklist of information that should be included in reports of global health estimates, with description of compliance and location of information for GBD 2016.

#	GATHER checklist item	Description of compliance	Reference
**Objectives and funding**			
1	Define the indicators, populations, and time periods for which estimates were made.	Narrative provided in paper and appendix describing indicators, definitions, and populations	Main text (Methods) and appendix
2	List the funding sources for the work.	Funding sources listed in paper	Summary (Funding)
**Data Inputs**			
For all data inputs from multiple sources that are synthesized as part of the study:			
3	Describe how the data were identified and how the data were accessed.	Narrative description of data seeking methods provided	Main text (Methods) and appendix
4	Specify the inclusion and exclusion criteria. Identify all ad-hoc exclusions.	Narrative about inclusion and exclusion criteria by data type provided; Adhoc exclusions in cause specific write ups	Main text (Methods) and appendix
5	Provide information on all included data sources and their main characteristics. For each data source used, report reference information or contact name/institution, population represented, data collection method, year(s) of data collection, sex and age range, diagnostic criteria or measurement method, and sample size, as relevant.	An interactive, online data source tool that provides metadata for data sources by component, geography, cause, risk, or impairment has been developed	Online data citation tools: http://ghdx.healthdata.org/gbd-2016
6	Identify and describe any categories of input data that have potentially important biases (e.g., based on characteristics listed in item 5).	Summary of known biases by cause included in appendix	Appendix
For data inputs that contribute to the analysis but were not synthesized as part of the study:			
7	Describe and give sources for any other data inputs.	Included in online data source tool	http://ghdx.healthdata.org/gbd-2016
For all data inputs:			
8	Provide all data inputs in a file format from which data can be efficiently extracted (e.g., a spreadsheet as opposed to a PDF), including all relevant meta-data listed in item 5. For any data inputs that cannot be shared due to ethical or legal reasons, such as third-party ownership, provide a contact name or the name of the institution that retains the right to the data.	Downloads of input data available through online tools, including data visualization tools and data query tools; input data not available in tools will be made available upon request	Online data visualization tools, data query tools, and the Global Health Data Exchange
**Data analysis**			
9	Provide a conceptual overview of the data analysis method. A diagram may be helpful.	Flow diagrams of the overall methodological processes, as well as cause-specific modelling processes, have been provided	Main text (Methods) and appendix

10	Provide a detailed description of all steps of the analysis, including mathematical formulae. This description should cover, as relevant, data cleaning, data pre-processing, data adjustments and weighting of data sources, and mathematical or statistical model(s).	Flow diagrams and corresponding methodological write-ups for each cause, as well as the databases and modelling processes, have been provided	Main text (Methods) and appendix
11	Describe how candidate models were evaluated and how the final model(s) were selected.	Provided in the methodological write-ups	Appendix
12	Provide the results of an evaluation of model performance, if done, as well as the results of any relevant sensitivity analysis.	Provided in the methodological write-ups	Appendix
13	Describe methods for calculating uncertainty of the estimates. State which sources of uncertainty were, and were not, accounted for in the uncertainty analysis.	Appendix	Appendix
14	State how analytic or statistical source code used to generate estimates can be accessed.	Appendix	http://ghdx.healthdata.org/gbd-2016-code
**Results and Discussion**			
15	Provide published estimates in a file format from which data can be efficiently extracted.	GBD 2016 results are available through online data visualization tools, the Global Health Data Exchange, and the online data query tool	Main text, and online data tools (data visualization tools, data query tools, and the Global Health Data Exchange)
16	Report a quantitative measure of the uncertainty of the estimates (e.g. uncertainty intervals).	Uncertainty intervals are provided with all results	Main text, appendix, and online data tools (data visualization tools, data query tools, and the Global Health Data Exchange)
17	Interpret results in light of existing evidence. If updating a previous set of estimates, describe the reasons for changes in estimates.	Discussion of methodological changes between GBD rounds provided in the narrative of the manuscript and appendix	Main text (Methods and Discussion) and appendix
18	Discuss limitations of the estimates. Include a discussion of any modelling assumptions or data limitations that affect interpretation of the estimates.	Discussion of limitations provided in the narrative of the main paper, as well as in the methodological write-ups in the appendix	Main text (Limitations) and appendix

Appendix 2: Flowchart of method

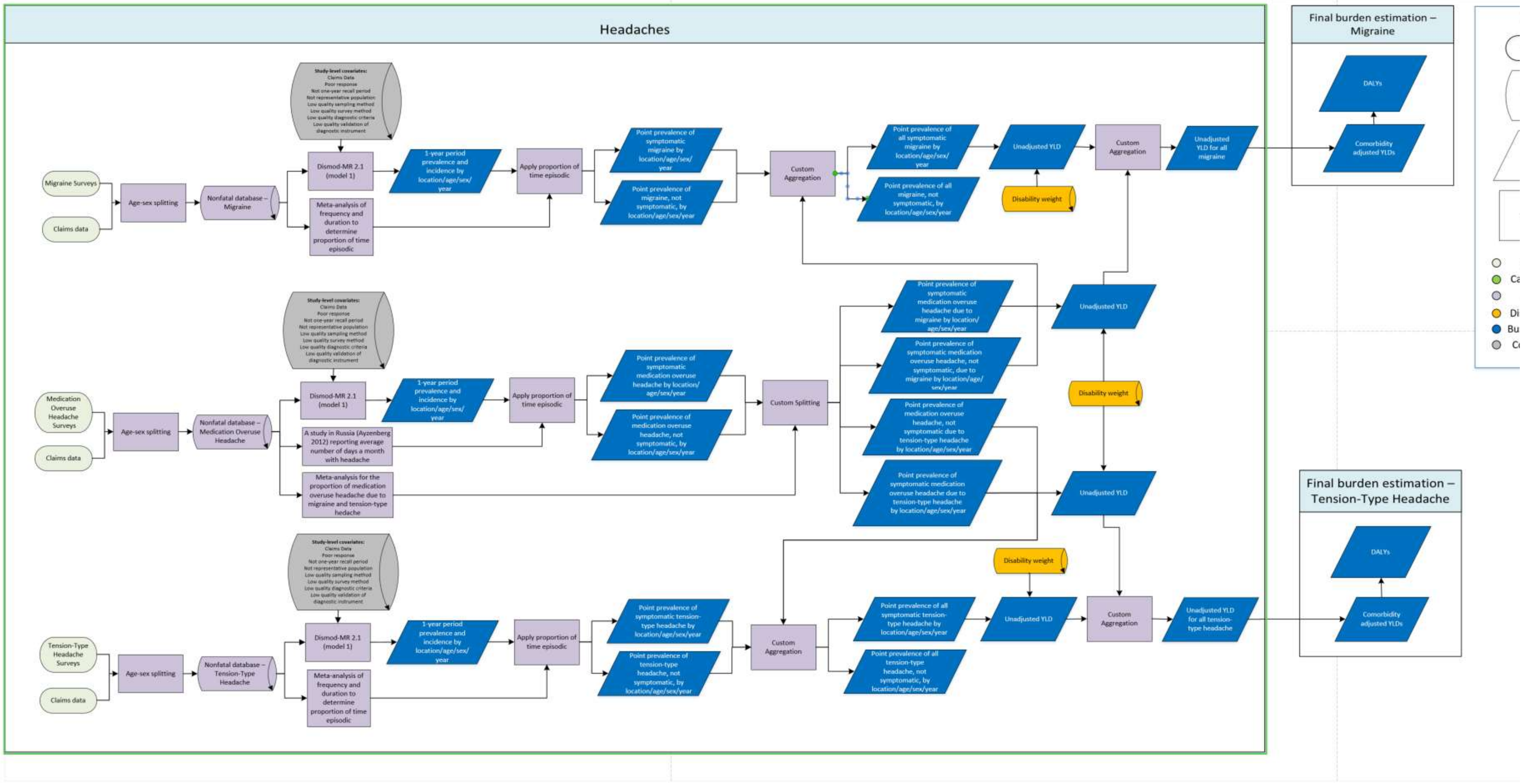

Appendix 3: Count of data sources used in nonfatal modeling of migraine (M), tension-type headache (TTH), and medication overuse headache (MOH) by 21 regions in 2016

	Incidence			Prevalence			Remission			Claims		
	M	TTH	MOH	M	TTH	MOH	M	TTH	MOH	M	TTH	MOH
East Asia	0	0	0	9	4	4	0	0	0	0	0	0
Southeast Asia	0	0	0	3	1	0	0	0	0	0	0	0
Oceania	0	0	0	0	0	0	0	0	0	0	0	0
Central Asia	0	0	0	0	0	1	0	0	0	0	0	0
Central Europe	0	0	0	3	2	0	0	0	0	0	0	0
Eastern Europe	0	0	0	4	2	2	0	0	0	0	0	0
High-income Asia Pacific	0	0	0	10	7	1	0	0	0	0	0	0
Australasia	0	0	0	4	4	0	0	0	0	0	0	0
Western Europe	4	1	0	37	20	15	0	0	7	0	0	0
Southern Latin America	0	0	0	2	2	0	0	0	0	0	0	0
High-income North America	1	0	0	14	5	0	1	0	0	3	3	0
Caribbean	0	0	0	0	0	0	0	0	0	0	0	0
Andean Latin America	0	0	0	4	2	0	0	0	0	0	0	0
Central Latin America	0	0	0	1	1	1	0	0	0	0	0	0
Tropical Latin America	0	0	0	5	7	1	0	0	0	0	0	0
North Africa and Middle East	0	0	0	16	7	2	0	0	0	0	0	0
South Asia	0	0	0	4	3	2	0	0	0	0	0	0
Central sub-Saharan Africa	0	0	0	0	0	0	0	0	0	0	0	0
Eastern sub-Saharan Africa	0	0	0	6	4	1	0	0	0	0	0	0
Southern sub-Saharan Africa	0	0	0	0	0	0	0	0	0	0	0	0
Western sub-Saharan Africa	0	0	0	4	1	0	0	0	0	0	0	0

Appendix 4: Studies used for estimating proportion of time in symptomatic state

Migraine

**Study and year**	**Country**	**Sex**	**Estimated proportion of time with migraine (95% CIs)**
Mavromichalis 1999[6]	Greece	M and F	0.041 (0.029-0.052)
Zivadinov 1985[4]	Croatia	M and F	0.121 (0.106-0.136)
Orji 1997[3]	Nigeria	M and F	0.081 (0.065-0.097)
Henry 1992[2]	France	F	0.095 (0.080-0.110)
Henry 1992[2]	France	M	0.095 (0.071-0.119)
Ensink 1994[1]	Germany	M and F	0.041 (0.0380.044)
Lipton 2001[5]	USA	F	0.088 (0.083-0.093)
Lipton 2001[5]	USA	M	0.081 (0.073-0.089)
Lavados 1997[7]	Chile	M	0.027 (0.009-0.045)
Lavados 1997[7]	Chile	F	0.022 (0.015-0.029)
Rao 2012[9]	India	M and F	0.042 (0.037-0.048)
Dent 2011[10]	Tanzania	M and F	0.034 (0.032-0.037)
Ayzenberg 2012[11]	Russia	M and F	0.090 (0.082-0.099)
Ertas 2012[12]	Turkey	M and F	0.283 (0.265-0.301)
Herekar unpublished[13]	Pakistan	M and F	0.185 (0.181-0.188)
Semiz 2013[8]	Turkey	M and F	0.034 (0.027-0.041)
**Pooled average**			**0.085 (0.056-0.114)**

F: Females M: Males

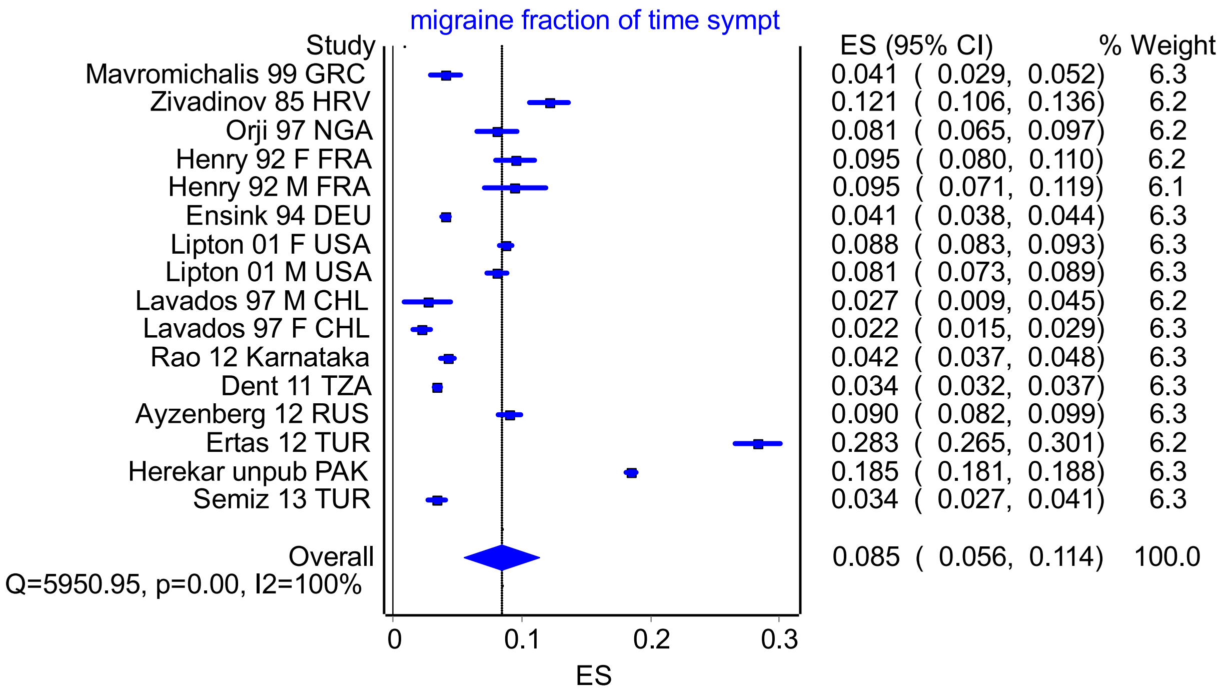

1. Ensink FB, Bautz M, Brüggenjürgen B, Hanekop GG. [Prevalence of migraine in Germany].*Schmerz*. 1994; 8(3): 155-61
2. Henry P, Michel P, Brochet B, Dartigues JF, Tison S, Salamon R, GRIM R. A nationwide survey of migraine in France: prevalence and clinical features in adults. Cephalalgia. 1992; 12(4): 229-237.
3. Orji GI, Iloeje SO. Childhood migraine in Nigeria--I: A community-based study. West Afr J Med. 1997; 16(4): 208-17.
4. Zivadinov R, Willheim K, Jurjevic A, Sepic-Grahovac D, Bucuk M, Zorzon M. Prevalence of migraine in Croatia: a population-based survey. Headache. 2001; 41(8): 805-12.
5. Lipton RB, Stewart WF, Diamond S, Diamond ML, Reed M. Prevalence and burden of migraine in the United States: data from the American Migraine Study II. Headache. 2001; 41(7): 646-57.

6. Mavromichalis I, Anagnostopoulos D, Metaxas N, Papanastassiou E. Prevalence of migraine in schoolchildren and some clinical comparisons between migraine with and without aura. Headache. 1999; 39(10): 728-36.
7. Lavados PM, Tenhamm E. Epidemiology of migraine headache in Santiago, Chile: a prevalence study. Cephalalgia. 1997; 17(7): 770-7.
8. Semiz M, Şentürk IA, Balaban H, Yağız AK, Kavakçı Ö. Prevalence of migraine and co-morbid psychiatric disorders among students of Cumhuriyet University. J Headache Pain. 2013; 14(1): 34.
9. Rao GN, Kulkarni GB, Gururaj G, Rajesh K, Subbakrishna DK, Steiner TJ, Stovner LJ. The burden of headache disorders in India: methodology and questionnaire validation for a community-based survey in Karnataka State. J Headache Pain. 2012; 13(7): 543-50.
10. Dent W, Stelzhammer B, Meindl M, Matuja WBP, Schmutzhard E, Winkler AS. Migraine attack frequency, duration, and pain intensity: disease burden derived from a community-based survey in northern Tanzania. Headache, 51(10): 1483–92.
11. Ayzenberg I, Katsarava Z, Sborowski A, Chernysh M, Osipova V, Tabeeva G, Yakhno N, Steiner TJ, Lifting the Burden. The prevalence of primary headache disorders in Russia: a countrywide survey. Cephalalgia. 2012; 32(5): 373-81.
12. Ertas M, Baykan B, Orhan EK, Zarifoglu M, Karli N, Saip S, Onal AE, Siva A. One-year prevalence and the impact of migraine and tension-type headache in Turkey: a nationwide home-based study in adults. *J Headache Pain*. 2012
13. Herekar A, Ahmed B, Effendi J, Latif U, Alvi Z, Steiner TJ on behalf of the Headache Research Foundation of Pakistan and Lifting The Burden: the Global Campaign against Headache. Pakistan Measuring the Burden of Headache in the General Population 2009 [Unpublished].

14. Rao GN, Kulkarni GB, Gururaj G, Rajesh K, Subbakrishna DK, Steiner TJ, Stovner LJ. The burden of headache disorders in India: methodology and questionnaire validation for a community-based survey in Karnataka State. J Headache Pain. 2012; 13(7): 543-50.

Tension-type headache (TTH)

Study and year	Country	Sex	Estimated proportion of time with TTH (95% CIs)
Lavados 1997[6]	Chile	M	0,014 (0,010-0,018)
Lavados 1997[6]	Chile	F	0,030 (0,023-0,036)
Levy 1983[5]	Zimbabwe	M and F	0,077 (0,068-0,085)
Lampl 2003[4]	Austria	M	0,038 (0,013-0,064)
Lampl 2003[4]	Austria	F	0,055 (0,033-0,076)
Isik 2006[7]	Turkey	M and F	0,041 (0,035-0,048)
Rao 2012[1]	India	M and F	0,013 (0,010-0,016)
Herekar unpublished[2]	Pakistan	M and F	0,116 (0,113-0,118)
Ayzenberg 2012[3]	Russia	M and F	0,035 (0,031-0,039)
Pooled average			0,047 (0,013-0,080)

F: Females, M: Males

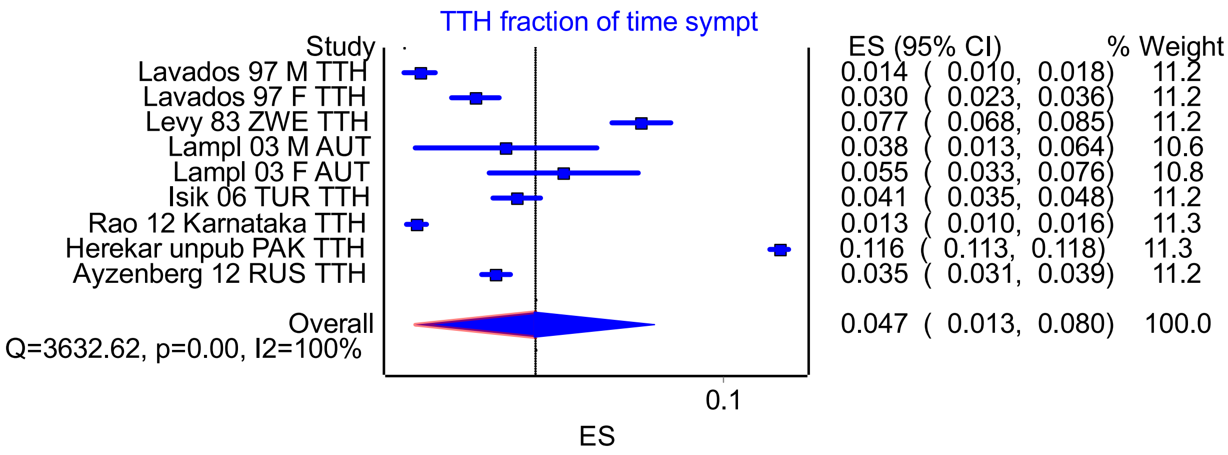

1. Rao GN, Kulkarni GB, Gururaj G, Rajesh K, Subbakrishna DK, Steiner TJ, Stovner LJ. The burden of headache disorders in India: methodology and questionnaire validation for a community-based survey in Karnataka State. J Headache Pain. 2012; 13(7): 543-50.
2. Herekar A, Ahmed B, Effendi J, Latif U, Alvi Z, Steiner TJ on behalf of the Headache Research Foundation of Pakistan and Lifting The Burden: the Global Campaign against Headache. Pakistan Measuring the Burden of Headache in the General Population 2009 [Unpublished].
3. Ayzenberg I, Katsarava Z, Sborowski A, Chernysh M, Osipova V, Tabeeva G, Yakhno N, Steiner TJ, Lifting the Burden. The prevalence of primary headache disorders in Russia: a countrywide survey. Cephalalgia. 2012; 32(5): 373-81.
4. Lampl C, Buzath A, Baumhackl U, Klingler D. One-year prevalence of migraine in Austria: a nation-wide survey. Cephalalgia. 2003; 23(4): 280-6.
5. Levy LM. An epidemiological study of headache in an urban population in Zimbabwe. Headache, 1983; 23(1): 2-9
6. Lavados PM, Tenhamm E. Epidemiology of migraine headache in Santiago, Chile: a prevalence study. Cephalalgia. 1997; 17(7): 770-7.
7. Isik U, Ersu RH, Ay P, Save D, Arman AR, Karakoc F, et al. Prevalence of headache and its association with sleep disorders in children. Pediatric neurology. 2007;36(3):146-51.
